# A highly expressed intestinal cysteine protease of *Ancylostoma ceylanicum* protects vaccinated hamsters from hookworm infection

**DOI:** 10.1371/journal.pntd.0007345

**Published:** 2019-04-22

**Authors:** Jason B. Noon, Erich M. Schwarz, Gary R. Ostroff, Raffi V. Aroian

**Affiliations:** 1 Program in Molecular Medicine, University of Massachusetts Medical School, Worcester, Massachusetts, United States of America; 2 Department of Molecular Biology and Genetics, Cornell University, Ithaca, New York, United States of America; McGill University, CANADA

## Abstract

**Background:**

Human hookworms (*Necator americanus*, *Ancylostoma duodenale*, and *Ancylostoma ceylanicum*) are intestinal blood-feeding parasites that infect ~500 million people worldwide and are among the leading causes of iron-deficiency anemia in the developing world. Drugs are useful against hookworm infections, but hookworms rapidly reinfect people, and the parasites can develop drug resistance. Therefore, having a hookworm vaccine would be of tremendous benefit.

**Methodology/Principal findings:**

We investigated the vaccine efficacy in outbred Syrian hamsters of three *A*. *ceylanicum* hookworm antigen candidates from two classes of proteins previously identified as promising vaccine candidates. These include two intestinally-enriched, putatively secreted cathepsin B cysteine proteases (AceyCP1, AceyCPL) and one small Kunitz-type protease inhibitor (AceySKPI3). Recombinant proteins were produced in *Pichia pastoris*, and adsorbed to Alhydrogel. Recombinant AceyCPL (rAceyCPL)/Alhydrogel and rAceySKPI3/Alhydrogel induced high serum immunoglobulin G (IgG) titers in 8/8 vaccinates, but were not protective. rAceyCP1/Alhydrogel induced intermediate serum IgG titers in ~60% of vaccinates in two different trials. rAceyCP1 serum IgG responders had highly significantly decreased hookworm burdens, fecal egg counts and clinical pathology compared to Alhydrogel controls and nonresponders. Protection was highly correlated with rAceyCP1 serum IgG titer. Antisera from rAceyCP1 serum IgG responders, but not nonresponders or rAceyCPL/Alhydrogel vaccinates, significantly reduced adult *A*. *ceylanicum* motility *in vitro*. Furthermore, rAceyCP1 serum IgG responders had canonical Th2-specific recall responses (IL4, IL5, IL13) in splenocytes stimulated *ex vivo*.

**Conclusions/Significance:**

These findings indicate that rAceyCP1 is a promising vaccine candidate and validates a genomic/transcriptomic approach to human hookworm vaccine discovery.

## Author summary

Hookworms are voracious, blood-feeding, soil-transmitted nematode parasites. Adult hookworms infect the small intestine, causing iron-deficiency anemia and other complications. Hookworms are among the most disabling parasites of the developing world. Drugs are useful for controlling hookworm disease. However, because people often get reinfected rapidly and parasites can develop drug resistance, a vaccine that provides long-term protection would improve control and help lead to eradication. At present, there is no licensed hookworm vaccine, and progress towards a vaccine has been limited. We identified a cysteine protease in the intestine of the human hookworm *Ancylostoma ceylanicum* that is among the most strongly expressed genes during blood feeding and that may help digest blood and be essential for hookworm survival. Vaccination of hamsters with this cysteine protease gave high levels of protection when antigen-specific antibodies in the blood were induced. These antigen-specific antibodies also made hookworms less mobile in culture. This cysteine protease is a promising candidate for further investigation as a human hookworm vaccine antigen.

## Introduction

Human hookworms (*Necator americanus*, *Ancylostoma duodenale*, and *Ancylostoma ceylanicum*) are soil-transmitted nematodes (STNs) that infect the small intestine and feed on blood [1]. Human STNs encompass three phylogenetically distant parasites: hookworms, large roundworms (*Ascaris lumbricoides*), and whipworms (*Trichuris trichiura*). Among human STNs, hookworms carry the highest disease burden [2]. In children, infection by hookworms causes significant growth stunting, cognitive deficiencies, malnutrition, iron-deficiency anemia and hypoproteinemia; in adults infection results in adverse birth outcomes (*e*.*g*., low birthweight babies) and reduced productivity [3–5]. It is estimated that hookworms infect ~500 million people worldwide [6], and although concentrated in Latin America, sub-Saharan Africa, and Southeast Asia, even people in impoverished regions of the United States (US) still get infected [1,7]. Once at 40% prevalence in the southern US (circa 1911), hookworm infections led to an estimated 43% reduction in future earnings of children infected and were responsible for 22% of the income gap and 50% of the literacy gap between North and South [8]. The elimination of hookworms via treatment campaigns, improved sanitation, education, and economic development undoubtedly had a major impact on the vitalization and success of the South today. Currently, hookworm disease is estimated to cause 4.1 million disability adjusted life years (DALYs) and US$139 billion in indirect economic losses each year [9]. Hookworms are the second most important parasitic cause of global anemia after malaria [10].

Mass drug administration (MDA) of benzimidazoles (albendazole, mebendazole) in school-aged children is the current control measure for hookworms [11,12]. From 1990–2013, MDA reduced hookworm prevalence by only 5.1%, compared to 25.5% reduction for *A*. *lumbricoides* [6]. Additionally, poor efficacy of mebendazole against hookworms is well known [13] and poor or reduced efficacy of albendazole against hookworms is being reported in multiple locations around the world (*e*.*g*., egg reduction rates as low as 0% in Ghana [14] and cure rates as low as 36% in Lao PDR [15]). Veterinary parasites phylogenetically and biologically similar to hookworms (*e*.*g*., the blood-feeding *Haemonchus contortus*) develop resistance to anthelmintic drugs frequently, rapidly, and broadly [16,17]. Water, sanitation and hygiene (WASH) is being explored as a control strategy to combine with MDA [18]. Improvements in WASH (water, sanitation, and hygiene), although important, are insufficient to tackle the enormous STN problem alone [18–20]. Having a vaccine to prevent infection from occurring in the first place would be of tremendous benefit.

Although it is widely accepted that a hookworm vaccine is needed [21], there is only a single phase 1 clinical trial underway testing two individual recombinant hookworm proteins formulated on the Th2 adjuvant Alhydrogel [22]. There are no other candidates in advanced preclinical development [23]. Because targeting infectious third staged larval (L3i) antigens carries the risk of triggering allergic reactions in previously exposed people [24], efforts are focused on adult stage antigens, namely an aspartic protease (APR1) and a glutathione S-transferase (GST1) [25]. Both APR1 and GST1 localize to the adult canine hookworm *Ancylostoma caninum* intestine (and non-intestinal tissues); these enzymes are thought to help digest hemoglobin and detoxify heme, respectively [25–28]. In canine and hamster models, recombinant protein immunogens from *A*. *caninum* gave 33–53% decreased hookworm burdens [26,27,29]. However, in a phase 1a trial, although rNaGST1 was safe and immunogenic in human volunteers [30], IgG had negligible neutralizing effect on rNaGST1 enzymatic activity, despite the observation that IgG from immunized mice were highly neutralizing [31]; these results suggest a decreased potential for this vaccine in human trials. It remains crucial that further and expanded efforts be undertaken to develop new hookworm vaccines. Development of hookworm vaccines, however, have been limited and lag far behind more concerted efforts, such as against malaria [23,32]. This may be in part because full genomes for human hookworms were formerly unavailable, which prevented large-scale reverse vaccinology [33] against hookworms. This situation has recently been addressed for all three species of human hookworms [34–36].

Genomics and transcriptomics for *A*. *ceylanicum* hookworm infections in hamsters is being used to identify new and potent vaccine antigen candidates [35]. Syrian hamsters are the only laboratory rodent permissive for the human hookworm life cycle, and *A*. *ceylanicum* infections in hamsters are an excellent model for hookworm infections in humans [37]. We previously identified two classes of *A*. *ceylanicum* genes, that are strongly expressed and upregulated during blood feeding, as encoding potential antigen candidates [35]: cathepsin B cysteine proteases (CPs) and small Kunitz-type protease inhibitors (SKPIs). Here, we explore the vaccine efficacy of two CPs and one SKPI (AceyCP1, AceyCPL, AceySKPI3) using the *A*. *ceylanicum* hookworm—hamster model system, and investigate functional and immunological aspects of protection with one of these vaccine candidates.

## Methods

### Ethics statement

Animal experimentation was carried out under protocols approved by the University of Massachusetts Medical School Institutional Animal Care and Use Committees (IACUC). All housing and care of laboratory animals used in this study conform to the NIH *Guide for the Care and Use of Laboratory Animals in Research* and all requirements and all regulations issued by the United States Department of Agriculture, including regulations implementing the Animal Welfare Act (P.L. 89–544).

### Animals

Male Syrian hamsters (strain HsdHan:AURA) were purchased from Envigo at 3 weeks of age and housed 4 hamsters per cage. Hamsters were provided with food and water *ad libitum*. Male hamsters were used for all studies because female hamsters are ~5-fold less susceptible to *A*. *ceylanicum* infection. Using females would require much larger numbers of animals to achieve adequate infection intensity, prevalence, and statistical significance.

### RNA-seq analysis

To assess intestinal versus non-intestinal gene expression for *A*. *ceylanicum* genes (including those encoding vaccine candidates in this study), we used RNA-seq data for *A*. *ceylanicum* that we and others had previously generated from whole worms and adult male intestine [35,38]. These data were a mixture of paired- and single-end reads with varying lengths. To make cross-comparisons of these data as unbiased as possible, we trimmed all read sets to have single-end 50-nt reads, using *quality_trim_fastq*.*pl* and the arguments "*-q 33 -u 50*". We quality-filtered RNA-seq reads by running Trimmomatic 0.36 with the following arguments: "*java -jar $TRIM/trimmomatic-0*.*36*.*jar SE -threads 7 -phred33 [input read FASTQ file] [output read FASTQ file] ILLUMINACLIP*:*[illumina adaptors sequence FASTA file]*:*2*:*30*:*10 LEADING*:*3 TRAILING*:*3 SLIDINGWINDOW*:*4*:*15 MINLEN*:*50*". The sequence file for Illumina adaptors included both sequences and reverse-complemented sequences for all of the following Illumina adaptor sequences from manufacturer's instructions: TruSeq Universal Adapter and TruSeq Adapter Index 1, 2, 3, 4, 5, 6, 7, 8, 9, 10, 11, 12, 13, 14, 15, 16, 18, 19, 20, 21, 22, 23, 25, and 27. We assayed expression levels against our previously published protein-coding gene/transcript set for *A*. *ceylanicum*, downloaded from the ParaSite database (release 6; *ftp://ftp.wormbase.org/pub/wormbase/parasite/releases/WBPS6/species/ancylostoma_ceylanicum/PRJNA231479/ancylostoma_ceylanicum.PRJNA231479.WBPS6.CDS_transcripts.fa.gz*) [39]. We quantitated gene expression from all of our quality-filtered *A*. *ceylanicum* RNA-seq data sets with Salmon 0.7.2 (*https*:*//github*.*com/COMBINE-lab/salmon/releases/download/v0*.*7*.*2/Salmon-0*.*7*.*2_linux_x86_64*.*tar*.*gz*), generating expression values in Transcripts Per Million (TPM) and estimating mapped read counts per gene [40]. For Salmon's index program, we used the arguments "*—no-version-check index—kmerLen 31—perfectHash—type quasi—sasamp 1*"; for Salmon's quant program, we used the arguments "*—libType A seqBias gcBias numBootstraps 100—geneMap [transcript-to-gene table]*", with "*—unmatedReads*" specifying the 50-nt single-end data. For gene annotations, we created new Pfam motif annotations with hmmscan from HMMER version 3.1b2 [41] and the Pfam 31.0 database, using the arguments "*—cut_ga -o /dev/null—tblout [table]*" which invoked reliably curated domain-specific thresholds; we also generated new InterPro motif annotations with *interproscan*.*sh* from InterProScan 5.18–57.0 [42], using the arguments "*-dp -hm -iprlookup -goterms*". Both Pfam and InterPro motifs were computed solely for the largest isoform of each gene's predicted protein products (downloaded from ParaSite release 6; *ftp://ftp.ebi.ac.uk/pub/databases/wormbase/parasite/releases/WBPS6/species/ancylostoma_ceylanicum/PRJNA231479/ancylostoma_ceylanicum.PRJNA231479.WBPS6.protein.fa.gz*); these largest isoforms were extracted with *get_largest_isoforms*.*pl* using the argument "*-t parasite*". All other gene annotations were taken from our previous work [35]. The Perl scripts *quality_trim_fastq*.*pl* and *get_largest_isoforms*.*pl* are available from *https*:*//github*.*com/SchwarzEM/ems_perl*.

### CDS cloning

Adult *A*. *ceylanicum* hookworms were collected from the small intestine of a day 22 post-inoculation (PI) hamster into a 1.5 mL microfuge tube, were rinsed three times with Milli-Q water, and then snap-frozen in liquid nitrogen and stored in -80°C. Tissue homogenization was performed in the same 1.5 mL microfuge tube on liquid nitrogen using a pre-chilled tapered flat end weighing spatula followed by a pre-chilled micropestle. Total nucleic acid was isolated with Nucleospin RNA kit (Machery-Nagel) according to the manufacturer’s instructions, except that on-column DNase treatment was omitted. The total nucleic acid was then treated with RNase-free DNase I (NEB) according to the manufacturer’s instructions. RNA was precipitated by addition of 1:10 vol 3 M sodium acetate and 2.5 vol ethanol with O/N storage in -20°C. The RNA pellet was washed twice with ethanol and then dissolved in 50 μL Milli-Q water. cDNA was synthesized with qScript cDNA SuperMix (Quantabio) according to the manufacturer’s instructions. PCR was performed with Platinum *Taq* DNA Polymerase High Fidelity (Invitrogen) according to the manufacturer’s instructions using the following primers: Aceys0154g3007cdsF and Aceys0154g3007cdsR (AceyCP1), Aceys0532g3038t1cdsF and Aceys0532g3038t1cdsR (AceyCPL), and Aceys0034g2829t1cdsF and Aceys0034g2829t1cdsR (AceySKPI3) (Table S1). The PCR products were purified with Monarch PCR and DNA Cleanup Kit (NEB) and were sequenced at GENEWIZ.

### Protein expression in *P*. *pastoris* X-33

The validated CDS sequences were sent to Genscript for *P*. *pastoris* codon-optimized DNA synthesis and the CDSs without native signal peptides (AceyCP1, nt 40–1,032; AceyCPL, nt 46–1,032; and AceySKPI3, nt 52–240) were subcloned into pPICZαA in-frame with yeast α-factor signal. During subcloning polyhistidine tags were added directly 5’ to the CDSs by PCR. The plasmids were linearized with SacI and transformed into *P*. *pastoris* X-33. Single transformed colonies (confirmed by colony PCR) were inoculated into 25 mL Buffered Glycerol-complex Medium (BMGY) and grown to an OD600 of 3.0. The 25 mL cultures were used to inoculate 0.5 L BMGY cultures at an OD600 of 1.0, and these cultures were grown to an OD600 of 3.0. The BMGY cultures were centrifuged and each was resuspended in 2 L of Buffered Methanol-complex Medium (BMMY) distributed into four 2 L baffled flasks, and these cultures were grown for 4 days. After 4 days of incubation, the BMMY cultures were centrifuged, and the clarified supernatants were collected.

### Protein purification from X-33 supernatants

rAceyCP1, rAceyCPL and rAceySKPI3 were purified from X-33 culture supernatants by immobilized metal affinity chromatography using a Ni resin and column (GenScript). Proteins bound to the resin were washed with Triton X-100 to reduce endotoxin levels to <1 EU/μg. The eluates were buffer exchanged into PBS (pH 7.4) by dialysis, and then filter sterilized with 0.22 μm Millex-GP Syringe Filters. Endotoxin levels were detected by ToxinSensor Gel Clot Endotoxin Assay Kit (GenScript). Protein concentrations were determined by Bradford assay using BSA as standard (GenScript).

### SDS-PAGE and Western blots

For SDS-PAGE, 4 μg of each protein was boiled for ~5 min in Pierce Lane Marker Reducing Sample Buffer (Thermo Fisher) and loaded into a 12% Tris-Glycine mini gel. Electrophoresis was run for ~2 hr at 100V in a Mini-PROTEAN Tetra Cell (Bio-Rad). Proteins were stained with Coomassie Blue, and the gels were imaged with a ChemiDoc XRS+ System with Image Lab Software (Bio-Rad). Molecular weights were estimated in Image Lab from the SDS-PAGE gels.

For Western blots, immediately after electrophoresis proteins were transferred to PVDF membranes using a Trans-Blot Turbo Transfer System with RTA Mini LF PVDF Transfer Kit (Bio-Rad). The membranes were blocked for 1.5 hr in blocking buffer (3% non-fat dry milk prepared in PBST). The blocked membranes were washed for 5 min twice with PBST, and then incubated for 1.5 hr in 6x-His Tag Monoclonal Antibody (HIS.H8) (Invitrogen) diluted 1:2,000 in blocking buffer. The membranes were then washed for 5 min 3 times with PBST, and then incubated for 1 hr in Goat anti-Mouse IgG (H+L) Secondary Antibody, HRP (Invitrogen) diluted 1:3,000 in blocking buffer. The membranes were washed for 5 min 3 times with PBST, and then incubated in the dark for 5 min in SuperSignal West Pico PLUS Chemiluminescent Substrate. The membranes were imaged with a ChemiDoc XRS+ System with Image Lab Software (Bio-Rad). Signal accumulation mode was used first to find the optimum exposure time (60 sec), and then the membranes were washed for 5 min with PBST and then incubated again in substrate. Finally, the membranes were imaged manually with the optimum exposure times.

### Vaccine trials

Vaccines were prepared fresh for each immunization on ice in a total volume of 1.8 mL (rAceyCPL/Alhydrogel, rAceySKPI3/Alhydrogel, rAceyCP1/Alhydrogel trial 1) or 2.6 mL (rAceyCP1/Alhydrogel trial 2) in sterile 5 mL microfuge tubes. Two hundred twenty-five μg (rAceyCPL/Alhydrogel, rAceySKPI3/Alhydrogel, rAceyCP1/Alhydrogel trial 1) or 325 μg (rAceyCP1/Alhydrogel trial 2) of immunogen was diluted up to 1.575 mL (rAceyCPL/Alhydrogel, rAceySKPI3/Alhydrogel, rAceyCP1/Alhydrogel trial 1) or 2.275 mL (rAceyCP1/Alhydrogel trial 2) in PBS (pH 7.4), and 225 μL ((rAceyCPL/Alhydrogel, rAceySKPI3/Alhydrogel, rAceyCP1/Alhydrogel trial 1) or 325 μL (rAceyCP1/Alhydrogel trial 2) of Alhydrogel (InvivoGen) was added. Immunogens were adsorbed to Alhydrogel according to the manufacturer’s instructions. For Alhydrogel control, 225 μL (rAceyCPL/Alhydrogel, rAceySKPI3/Alhydrogel, rAceyCP1/Alhydrogel trial 1) or 325 μL (rAceyCP1/Alhydrogel trial 2) of Alhydrogel was added to 1.575 mL (rAceyCPL/Alhydrogel, rAceySKPI3/Alhydrogel, rAceyCP1/Alhydrogel trial 1) or 2.275 mL (rAceyCP1/Alhydrogel trial 2) of PBS (pH 7.4). Final doses were 25 μg of protein and 250 μg of aluminum content (per dose).

Hamsters were injected subcutaneously (SC) with insulin syringes (BD) three times with two-week intervals in the scruff of the neck with 200 μL of vaccine. One week after the final immunization, hamsters were separated into individual cages, and the individual cages were randomly arranged on the shelves. Twelve days after the final immunization, peripheral blood was collected by saphenous venipuncture using PrecisionGlide 20 G x 1” hypodermic needles (BD) and SAFE-T-FILL Capillary Blood Collection Tubes–Serum (RAM Scientific). Blood was allowed to clot for at least 30 min at room temperature before centrifugation. The collected serum was stored in -20°C until ELISA. Thirteen days after the final immunization, hamsters were weighed and blood was collected again, but into SAFE-T-FILL Capillary Blood Collection Tubes–EDTA (RAM Scientific). Blood hemoglobin concentrations (g/dL) were measured with a STAT-Site M Hgb Hemoglobin Analyzer and Test Cards (Stanbio).

Exactly two weeks after the final immunization hamsters were inoculated with ~150 *A*. *ceylanicum* L3i by oral gavage. L3i were obtained by coproculture of feces collected from infected hamsters, and had been stored in the dark at room temperature for <2 weeks in BU buffer (50 mM Na_2_HPO_4_, 22 mM KH_2_PO_4_, 70 mM NaCl, pH 6.8) plus PSF (100 U/mL penicillin; 100 μg/mL streptomycin; 0.25 μg/mL amphotericin B) before inoculations. This *A*. *ceylanicum* line was originally obtained from Dr. John Hawdon at George Washington University. On day 20 PI, hamsters were weighed and blood hemoglobin concentrations measured as before. On day 22 PI, hamsters were placed on fecal collection wires overnight. Two layers of moistened paper towels were placed in the bottoms of the cages underneath the fecal collection wires. On day 23 PI, hamsters were euthanized by CO_2_ overdose and cervical dislocation (according to IACUC protocol). Small intestines were removed, longitudinally sectioned, and incubated in Hank’s Balanced Salt Solution (HBSS; Thermo Fisher) for 45 min at 37°C, 5% CO_2_. Hookworm burdens were counted under a stereomicroscope. Fecal pellets were collected from the cage of each hamster, and FECs were measured using a McMaster chamber (Hausser Scientific).

### Serum ELISA

Immunogens were coated overnight at 4°C onto Nunc MaxiSorp flat-bottom 96-well plates at 5 μg/ml in carbonate/bicarbonate (100 mM), pH 9.6 coating buffer (100 μL/well). Wells were washed three times with PBST (200 μL/well), and then blocked for 1.5 hr in blocking buffer (5% non-fat dry milk in PBST) (200 μL/well) at room temperature. Wells were washed two times with PBST (200 μL/well), and then hamster sera serially diluted in blocking buffer was incubated (100 μL/well) for 1.5 hr at room temperature. Wells were washed three times with PBST (200 μL/well), and then Peroxidase AffiniPure Goat Anti-Syrian Hamster IgG (H+L) (Jackson ImmunoResearch) was diluted 1:5,000 in blocking buffer and incubated in the wells (100 μL/well) for 1.5 hr at room temperature. Wells were washed three times with PBST (200 μL/well), and 100 μL of 1-Step Ultra TMB-ELISA Substrate Solution (Thermo Fisher) was incubated in the wells for 30 min. Then 100 μL of sulfuric acid (2 M) was added to the wells, and A450 was measured with a Tecan Safire plate reader.

In multiple pilot experiments, we tested Rabbit anti-Syrian hamster IgM-HRP (Rockland), Goat anti-Mouse IgA-HRP (Thermo Fisher; [43]) and Goat anti-Mouse IgE-HRP (Thermo Fisher) in ELISAs to soluble *A*. *ceylanicum* hookworm extract (HEX; [43]) using serum from infected and drug-cleared hamsters. Anti-Syrian hamster IgM-HRP gave extremely high background to HEX with serum from uninfected hamsters. Neither anti-Mouse IgA-HRP or anti-Mouse IgE-HRP reacted to HEX with serum from infected hamsters, while Goat anti-Syrian hamster IgG (mentioned above) reacted strongly to HEX only with serum from infected hamsters. Thus, only Goat anti-Syrian hamster IgG was useful for serum ELISAs.

### *In vitro* hookworm antiserum toxicity assays

*A*. *ceylanicum* adult hookworms were collected from the small intestines of initially naïve hamsters on day 17 PI. Small intestines were longitudinally sectioned and incubated for 2 hr in HBSS pre-warmed at 37°C in a mini Baermann Funnel apparatus. Every 20–30 min the small intestines were moved around with forceps. The HBSS containing motile hookworms that had migrated through the wire mesh and settled at the bottom of the funnel was poured into a petri dish, and the motile hookworms were hand-picked with a worm picker into Milli-Q water, and rinsed three times. Hookworms were hand-picked into individual wells of a 96-well plate (two hookworms per well) containing 50 μL of HCM with 50% heat-inactivated fetal bovine/calf serum [44] replaced by hamster serum (mHCM: 49.5% RPMI 1640 Medium containing L-glutamine without Phenol Red Indicator [Thermo Fisher]; 49.5% hamster serum; 1% 100X PSG [100 U/mL penicillin; 100 μg/mL streptomycin; 0.292 mg/mL L-glutamine; Thermo Fisher]). Each hamster serum group included three wells (two hookworms/well for a total of six hookworm adults scored per group), and the average motility for the three wells was calculated using a standard 3–0 motility index assay (3 = highly motile; 2 = less motile; 1 = motile only when stimulated by touch; 0 = immotile) [45–47]. Motility was monitored for 76 hr (once per day). To address any concerns about subjectivity, each replicate well for each condition was randomized in the setup so that the investigator was blinded as to which well contained which condition during the scoring process.

### *Ex vivo* mitogenic splenocyte cytokine assay

Another set of hamsters was vaccinated with rAceyCP1/Alhydrogel and Alhydrogel control exactly as before in the vaccine trials. Serum IgG responses were evaluated by ELISA as before using A450 readings at 1:100 serum dilutions. Two weeks after the final immunization hamsters were euthanized as before. Spleens were removed with ethanol-sterilized forceps and transferred to 5 mL DMEM-10 cell culture medium (89.5% DMEM [Dulbecco's Modification of Eagle's Medium; Mediatech]; 9.5% fetal calf serum [Thermo Fisher]; 1% 100X PSG; sterilized with 0.22 μm filter; stored in 4°C) in 60-mm petri dish on ice. Each spleen was cut into three pieces with ethanol-sterilized surgical scissors. Each spleen piece was smashed individually between two frosted microscope slides and rinsed back into the petri dish, removing any remaining solid tissue left on the slide with forceps. Each spleen material in DMEM-10 was passed through syringe needle series of 18, 22, and 26 G (BD) to prepare the splenocyte suspension that was collected into a 15-mL conical centrifuge tube pre-chilled on ice. Splenocyte suspensions were centrifuged at 1,500 rpm for 5 min at 4°C. The supernatant was discarded and cell pellet resuspended in 1 mL ACK Lysing Buffer (Thermo Fisher) and incubated for 10 min at room temperature. Immediately after, 9 mL of DMEM-10 was added and then centrifuged as before. Each splenocyte suspension was resuspended in 2 mL DMEM-10, and 1 mL was transferred into each of 2 different wells of a 48-well plate. For each splenocyte suspension, 25 μg of rAceyCP1 was added to one well and PBS (pH 7.4) to the other well. Splenocytes were stimulated for 48 hr at 37°C, 5% CO_2_.

Stimulated splenocytes were transferred to 1.5 mL microfuge tubes and centrifuged as before. Supernatants were discarded, cell pellets were resuspended in lysis buffer and vortexed, and RNA was isolated as before. Either 10 μg (γ-actin, IFN-γ, IL17A, IL10, and TGF-β) or 50 μg (IL4, IL5, IL13, and IL21) of RNA (as determined in primer efficiency tests) was used as template for qRT-PCR using qScript One-Step SYBR Green qRT-PCR Kit (Quantabio) according to the manufacturer’s instructions. Protocol: 49°C for 10 min, 95°C for 5 min, 35 cycles of 95°C for 15 sec and 60°C for 45 sec. Amplification specificities were verified by melting curve analysis. Melting curve protocol: 95°C for 1 min, 55°C for 10 sec and a slow temperature ramp from 55 to 95°C. The primer sets (Table S1; [48]) were pre-validated with mean ± S.D. amplification factors of 2.0 ± 0.3 in primer efficiency tests using the same sample type. qRT-PCR was performed on an Eppendorf Mastercycler RealPlex^2^. Four technical replicates were included for every primer set on each RNA sample. Minus reverse transcriptase reactions were also run on every RNA sample for every primer set, and this indicated that the RNA samples had undetectable levels of gDNA. γ-actin was used as reference. We had hoped to look at even more cytokines by qRT-PCR, but we did not have RNA for all biological replicates. Data were analyzed using the 2^-ΔΔCT^ method [49].

### Statistical analysis

All data analyses were plotted in Prism 7 (GraphPad Software). For serum IgG endpoint titers, plotted are the inverses of the final serum dilutions for each serum sample from each hamster within each vaccine group that gave an A450 reading that is >3 S.D. from the mean A450 reading for naïve hamster sera (n = 8) at the same dilution. For hookworm burden, mean indicates the mean hookworm burden amongst all hamsters in each vaccine group. For FECs (eggs/g), mean indicates the egg count per group from all cages in the group at a given time point. For Δweight and Δhemoglobin, mean indicates the mean Δweight and Δhemoglobin amongst all hamsters in each vaccine group. For motility index, mean indicates the mean motility amongst all wells (2 hookworms/well, 3 wells/group) in the group at a given time point. For serum IgG A450, plotted are the raw A450 readings for each serum sample from each hamster per vaccine group at 1:100 dilution with average Alhydrogel background subtracted. For relative fold change in mRNA, mean indicates the mean relative fold change in mRNA in splenocytes amongst all hamsters in each vaccine group.

Statistical 2-sample comparisons between experimental and control vaccine groups were carried out by one-tailed Mann-Whitney, with the hypothesis that successful vaccination will result in decreases in infection parameters (worm burdens, fecal egg counts) and improvements in measurements of sequelae (weight, hemoglobin). Nearly identical statistical results were obtained using student’s t test. All comparisons between multiple experimental groups with control (*i*.*e*., Alhydrogel alone) group were carried out using one-way ANOVA with Dunnett’s post-hoc test, comparing each group to control. Significant correlations in linear regression analyses (*i*.*e*., if slopes are significantly non-zero) were determined by F test.

## Results

### Production of three vaccine antigen candidates

Transcriptomic analyses revealed that the hookworm *A*. *ceylanicum* upregulates proteases and protease inhibitors during infection, which are likely to be important for successful parasitism [35]. Proteases are thought to help hookworms dig through their host's tissues, destroy proteins needed for the host's immune response, and digest proteins in the host's blood [50]. Protease inhibitors are thought to block host proteases needed for the immune response as well [51]. These proteases and protease inhibitors therefore defined an initial set of vaccine candidates and included putatively secreted cathepsin B-like proteases (CPs), which have homologs in other strongylids, and a previously undescribed family of putatively secreted small Kunitz-type protease inhibitors (SKPIs) that are strongly upregulated in adult hookworms [35].

**Fig 1:**
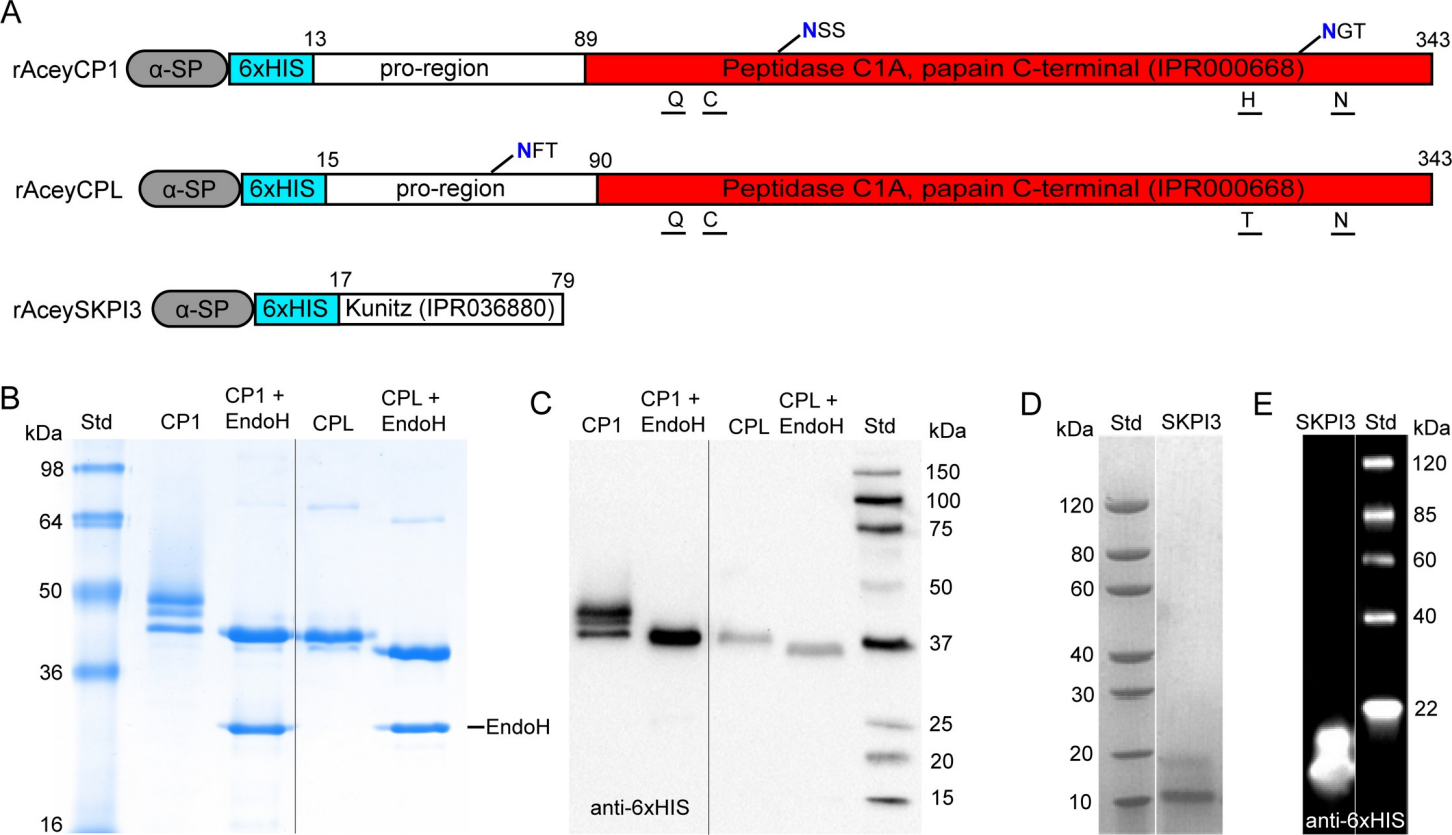
Production of secreted rAceyCP1, rAceyCPL and rAceySKPI3 from *Pichia pastoris*. (A) Schematic diagrams of rAceyCP1, rAceyCPL and rAceySKPI3. Amino acid positions are indicated above. Putative active site residues are indicated below. N-glycosylation sites are indicated above at their approximate locations with the glycosylated asparagine colored blue. AceyCPL and AceyCP1 share 54% amino acid identity. (B) SDS-PAGE of untreated and endoglycosidase (Endo) H-treated rAceyCP1 and rAceyCPL (Coomassie Blue image). (C) Western blot of untreated and Endo H-treated rAceyCP1 and rAceyCPL (grayscale image). (D) SDS-PAGE analysis of rAceySKPI3 (grayscale image). (E) Western blot of rAceySKPI3 (inverted grayscale image).

We successfully cloned full-length cDNAs for *A*. *ceylanicum* CP1 (rAceyCP1; AceyCP1 [genomic name, Acey_s0154.g3007]), AceyCPL (rAceyCPL [Acey_s0532.g3038]), and AceySKPI3 (rAceySKPI3 [Acey_s0034.g2829]) (Fig 1A). We expressed the proteins in *Pichia pastoris* and purified them using standard protocols (Fig 1B–1E; see Methods for details). *P*. *pastoris* expression was used for previous hamster-hookworm vaccine trials [52,53]. Since the hookworm intestine is a key target of protective vaccine antigens, we examined expression of these genes in published male hookworm intestinal transcriptomic data (Table S2) [38]. AceyCP1 is very strongly expressed in the male intestine at 11,600 transcripts per million (TPM), making it 1.2% of all transcripts in that tissue. AceyCPL and AceySKPI3 are present but considerably more weakly expressed in the male hookworm intestine at 52 TPM and 1.7 TPM respectively. Neither AceyCP1 nor AceyCPL are expressed in L3i (0.15 and 0 TPM respectively). AceySKPI3 has modest expression in L3i (14 TPM), but this is less than or comparable to expression levels for the *A*. *ceylanicum* homologs of APR1 and GST1 (AceyAPR1 [Acey_s0242.g3404], 744 TPM; AceyGST1 [Acey_s0110.g143], 44 TPM), which in *N*. *americanus* are considered acceptable vaccine candidates.

By SDS-PAGE and Western blot, three bands were observed for rAceyCP1 at ~42, ~44 and ~46 kDa (Fig 1B and 1C), two bands were observed for rAceyCPL at ~40 and ~42 kDa (Fig 1B and 1C), and two bands were observed for rAceySKPI3 at ~12 and ~19 kDa (mostly single band at ~12 kDa; Fig 1D and 1E). AceyCP1 and AceyCPL are proenzymes containing N-terminal pro-regions and a CP domain (Fig 1A). Two N-glycosylation sites were identified in AceyCP1 with NetNGlyc 4.0 (*http*:*//www*.*cbs*.*dtu*.*dk/services/NetNGlyc*) [54], both of which are located in its CP domain near putative active site residues (Fig 1A). One N-glycosylation site was identified in AceyCPL within its pro-region (Fig 1A). These predicted N-glycosylation sites match up with the observed banding patterns (Fig 1B and 1C). NetNGlyc 4.0 did not identify any potential N-glycosylation sites in AceySKPI3 (Fig 1A). Treatment of rAceyCP1 and rAceyCPL with endoglycosidase (Endo) H resulted in shifts to a single ~42-kDa band for rAceyCP1 and a single ~40-kDa band for rAceyCPL (Fig 1B and 1C). According to these results, rAceyCP1 consists of unglycosylated, monoglycosylated, and biglycosylated forms, while rAceyCPL consists of unglycoslyated and monoglycosylated forms. The weak ~19-kDa upper band in rAceySKPI3 could be a form of dimer or a monomer with other post-translational modification(s). Densitometric analysis determined all three recombinant proteins to be >95% pure.

### rAceyCPL/Alhydrogel and rAceySKPI3/Alhydrogel induce consistently high serum IgG titers but are not protective

**Fig 2:**
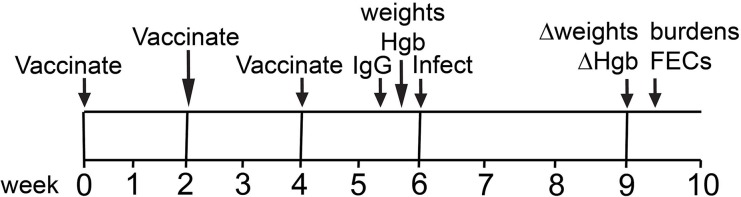
Vaccine trial timeline. Abbreviations—IgG: serum immunoglobulin G titers determined by ELISA; Hgb: blood hemoglobin concentration determined; weights: hamsters weighed; Δweights, change in weights after hookworm infection calculated after second weight taken; ΔHgb, change in blood hemoglobin concentrations after hookworm infection calculated after second hemoglobin measurement taken; FECs, overnight fecal egg counts determined.

**Fig 3:**
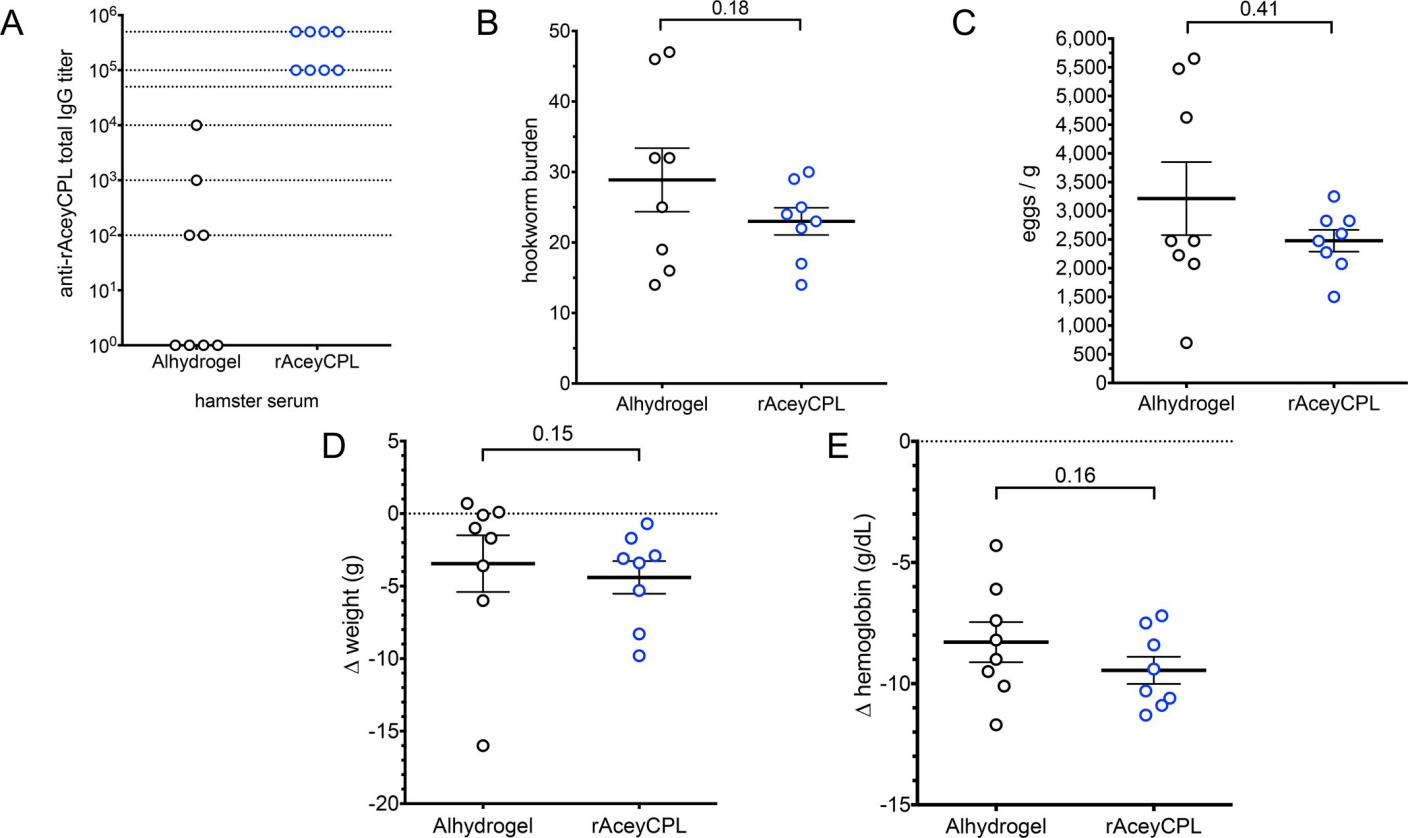
rAceyCPL/Alhydrogel vaccine trial. (A) rAceyCPL serum IgG titers in Alhydrogel controls and rAceyCPL/Alhydrogel vaccinates. Dotted lines indicate the inverse of serum dilutions tested. (B) Comparison of mean hookworm burden between rAceyCPL/Alhydrogel and Alhydrogel control vaccinates. (C) Comparison of mean FECs between rAceyCPL/Alhydrogel and Alhydrogel control vaccinates. (D) Comparison of mean change in weight after hookworm infection between rAceyCPL/Alhydrogel and Alhydrogel control vaccinates. (E) Comparison of mean change in hemoglobin concentration after hookworm infection between rAceyCPL/Alhydrogel and Alhydrogel control vaccinates. Numbers above bars indicate P values for comparison. (B-E) Error bars here and all figures indicate standard error.

rAceyCPL was adsorbed to Alhydrogel and injected SC into Syrian hamsters three times at two-week intervals (Fig 2, timeline). Twelve days after the final immunization (Fig 2), rAceyCPL serum IgG titer was determined by indirect enzyme-linked immunosorbent assay (ELISA). All rAceyCPL/Alhydrogel vaccinates (8/8) had serum IgG titers above background in Alhydrogel controls ranging from 100,000 to ≥500,000 (Fig 3A). Hamsters were infected with ~150 *A*. *ceylanicum* third stage infective larvae (L3i) exactly two weeks after the final immunization (Fig 2). At day 23 post-inoculation (PI), the number of hookworms in the small intestines (hookworm burden) were counted at necropsy, and feces were collected for fecal egg counts (FECs; hookworm eggs shed per g of feces). Mean hookworm burden and FEC were statistically similar between rAceyCPL/Alhydrogel vaccinates and Alhydrogel controls (Fig 3B and 3C), indicating that parasitism was unaffected by vaccination. As markers for the clinical pathology caused by hookworm infection, changes in weight and hemoglobin (Δweight and Δhemoglobin) were evaluated (on day 20 post-inoculation (PI) prior to necropsy; Fig 2). Consistent with hookworm burden and FEC, mean Δweight and Δhemoglobin were statistically similar between rAceyCPL/Alhydrogel vaccinates and Alhydrogel controls (Fig 3D and 3E), indicating that clinical pathology was also unaffected by vaccination.

**Fig 4:**
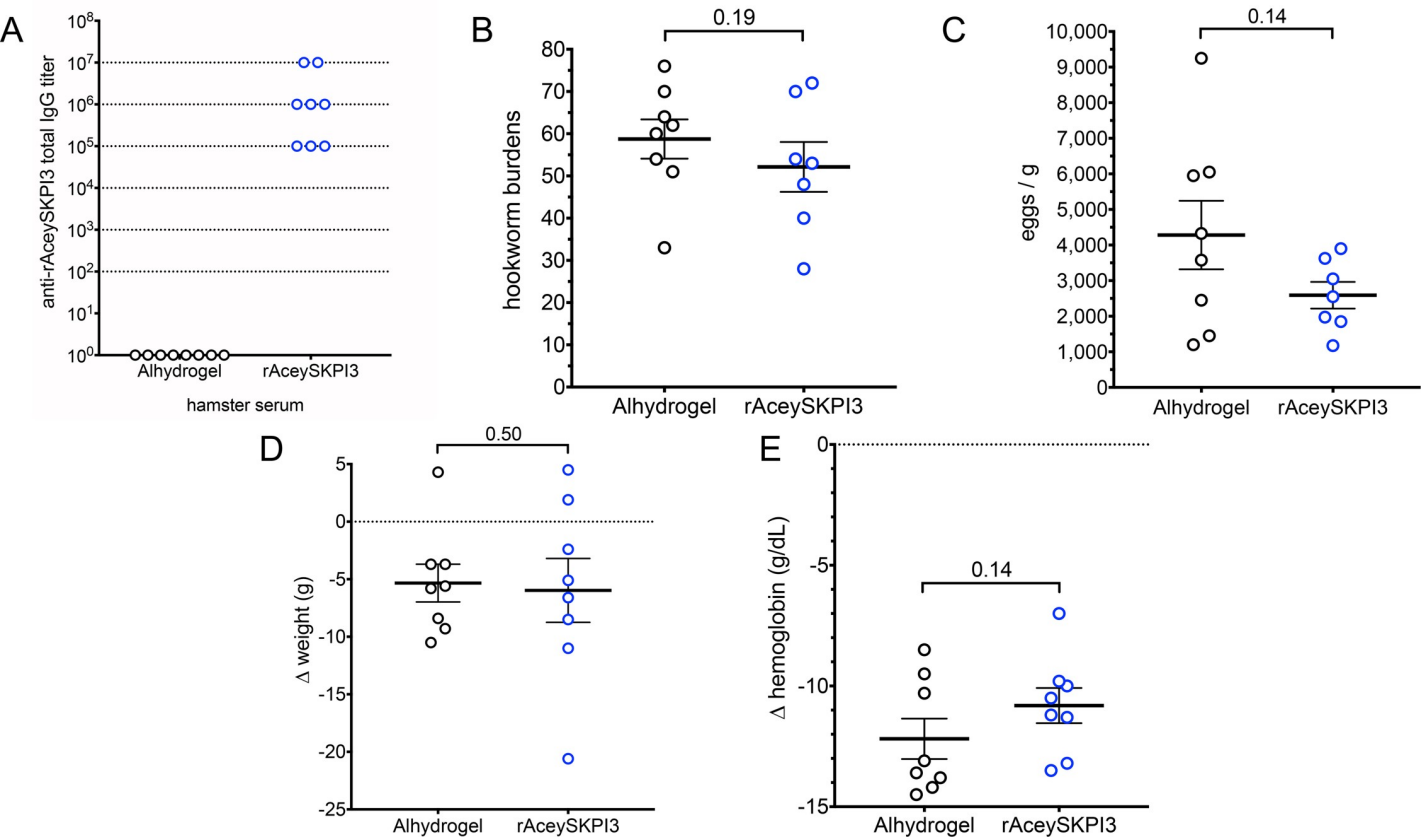
rAceySKPI3/Alhydrogel vaccine trial. (A) rAceySKPI3 serum IgG titers in Alhydrogel controls and rAceySKPI3/Alhydrogel vaccinates. Dotted lines indicate the inverse of serum dilutions tested. (B) Comparison of mean hookworm burden between rAceySKPI3/Alhydrogel and Alhydrogel control vaccinates. (C) Comparison of mean FEC between rAceySKPI3/Alhydrogel and Alhydrogel control vaccinates. (D) Comparison of mean change in weight after hookworm infection between rAceySKPI3/Alhydrogel and Alhydrogel control vaccinates. (E) Comparison of mean change in hemoglobin concentration after hookworm infection between rAceySKPI3/Alhydrogel and Alhydrogel control vaccinates. (B, C) For rAceySKPI3/Alhydrogel, n = 7 as 1 vaccinate died on day 21 PI (*i*.*e*., one day after obtaining Δweight (D) and Δhemoglobin (E); hookworm burden and FEC were determined on day 23 PI).

All rAceySKPI3/Alhydrogel vaccinates (8/8) had serum IgG titers above background in Alhydrogel controls ranging from 100,000 to, remarkably, ≥10,000,000 (Fig 4A). However, as with rAceyCPL/Alhydrogel, rAceySKPI3/Alhydrogel vaccination did not significantly alter hookworm parasitism (Fig 4B and 4C), or clinical pathology (Fig 4D and 4E) compared to Alhydrogel controls. Thus, these results indicate that although both rAceyCPL/Alhydrogel and rAceySKPI3/Alhydrogel induce high serum IgG titers above background with 100% responder rates, these antigens are not protective.

### rAceyCP1/Alhydrogel induces intermediate serum IgG titers and responders are protected against hookworm infection

**Fig 5:**
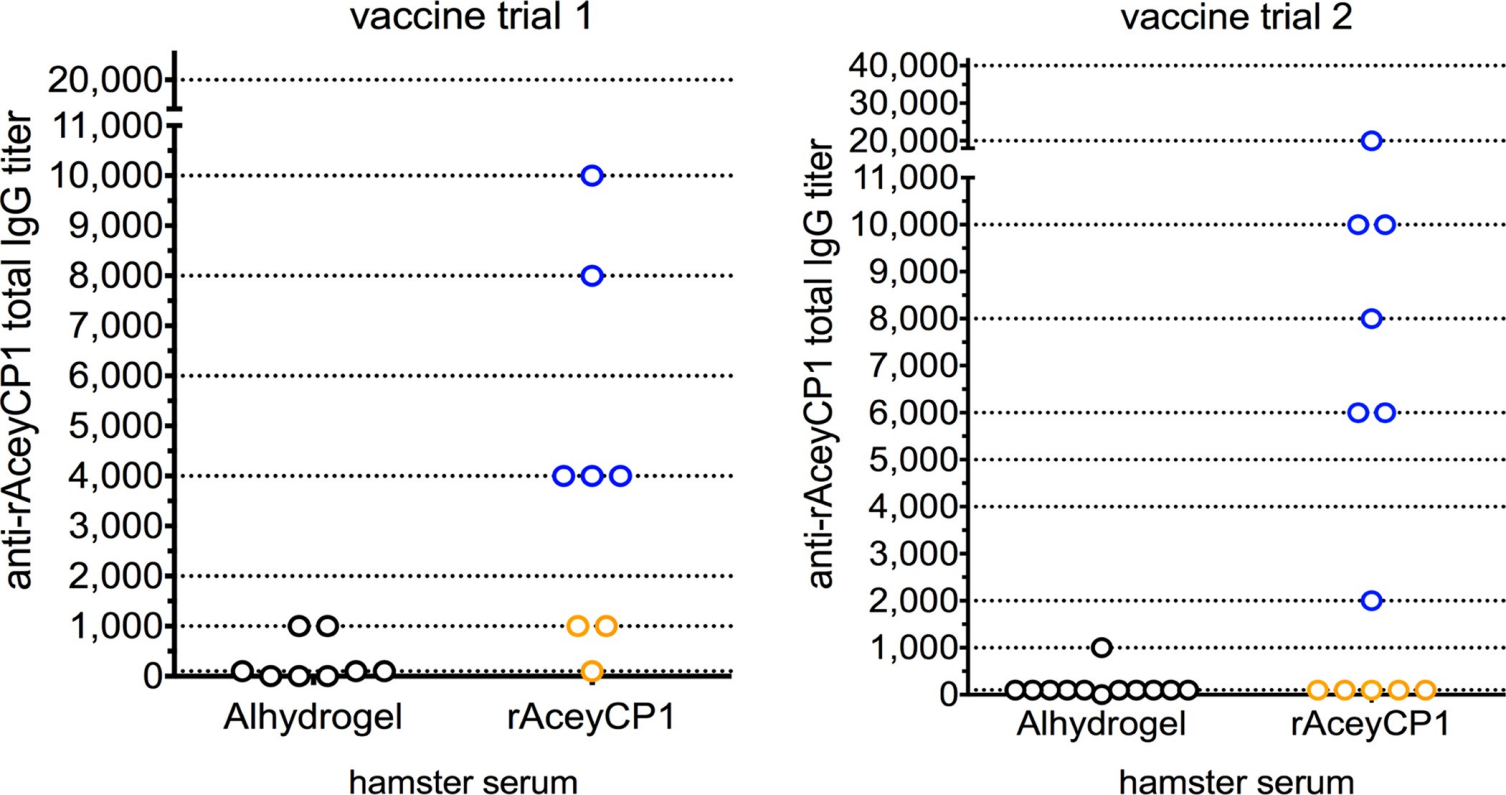
rAceyCP1 serum IgG titers in rAceyCP1/Alhydrogel and Alhydrogel control vaccinates. Blue dots indicate rAceyCP1 serum IgG responders (IgG titers above background levels found in Alhydrogel controls). Orange dots indicate rAceyCP1 serum IgG nonresponders. Dotted lines are indicated at the inverses of each serum dilution tested.

**Fig 6:**
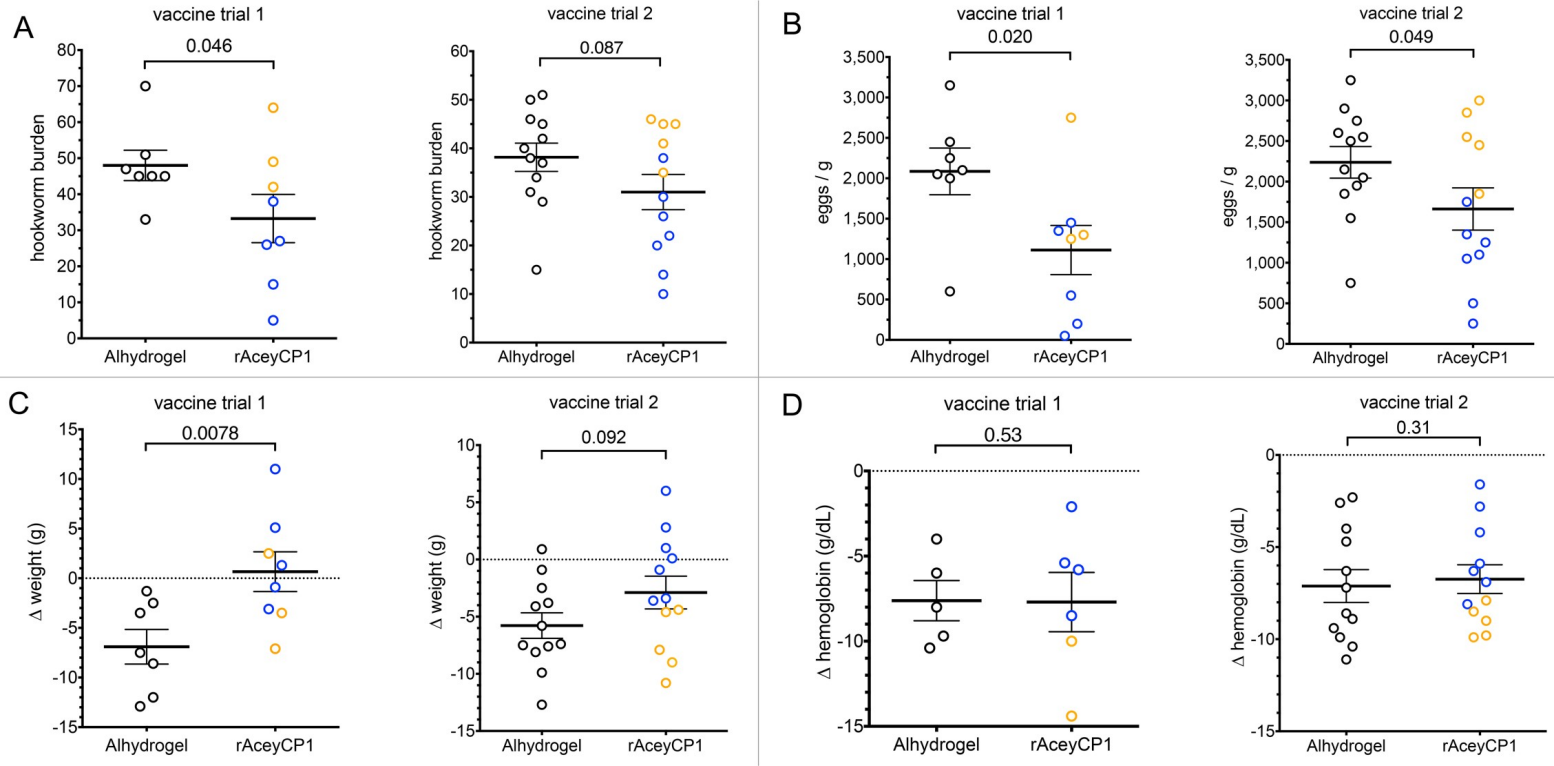
Efficacy results for rAceyCP1/Alhydrogel vaccine trials 1 and 2 with all animals combined. (A) Comparison of mean hookworm burden between rAceyCP1/Alhydrogel and Alhydrogel control vaccinates. Blue dots indicate serum IgG responders, orange dots indicate serum IgG nonresponders (see Fig 5). (B) Comparison of mean FEC between rAceyCP1/Alhydrogel and Alhydrogel control vaccinates. (C) Comparison of mean change in weight after hookworm infection between rAceyCP1/Alhydrogel and Alhydrogel control vaccinates. (D) Comparison of mean change in hemoglobin concentration after hookworm infection between rAceyCP1/Alhydrogel and Alhydrogel control vaccinates.

Two independent vaccine trials were conducted for rAceyCP1, the first with eight animals per group and the second with 12 animals per group. In trial 1, 5/8 vaccinates (62.5%) had rAceyCP1-specific serum IgG titers above background in Alhydrogel controls ranging from 4,000–10,000 (Fig 5). In trial 2, 7/12 vaccinates (58.3%) had rAceyCP1 serum IgG titers above background in Alhydrogel controls ranging from 2,000–20,000 (Fig 5). In trial 1 and trial 2, hookworm burdens were decreased in rAceyCP1/Alhydrogel vaccinates compared to Alhydrogel control vaccinates by a mean of 31% and 19% respectively, achieving statistical significance in trial 1 (Fig 6A) and nearly achieving statistical significance in trial 2 (Fig 6A). Significant decreases in FECs were seen in both trials (Fig 6B). In trial 1 and trial 2, respectively, FECs were significantly decreased in rAceyCP1/Alhydrogel vaccinates compared to Alhydrogel control vaccinates by a mean of 46% and 26%.

Vaccination with rAceyCP1 also generally led to improvements of hookworm sequelae. Δweight was signficantly improved in rAceyCP1/Alhydrogel vaccinates by 7.6 g (Syrian hamsters weigh around 115 g at this age; ~12 weeks) compared to Alhydrogel controls in trial 1 (Fig 6C), and there was a trend of 2.8 g toward improved Δweight in trial 2 (Fig 6C). The Δhemoglobin was statistically similar between rAceyCP1/Alhydrogel vaccinates and Alhydrogel controls in both trials (Fig 6D).

**Fig 7:**
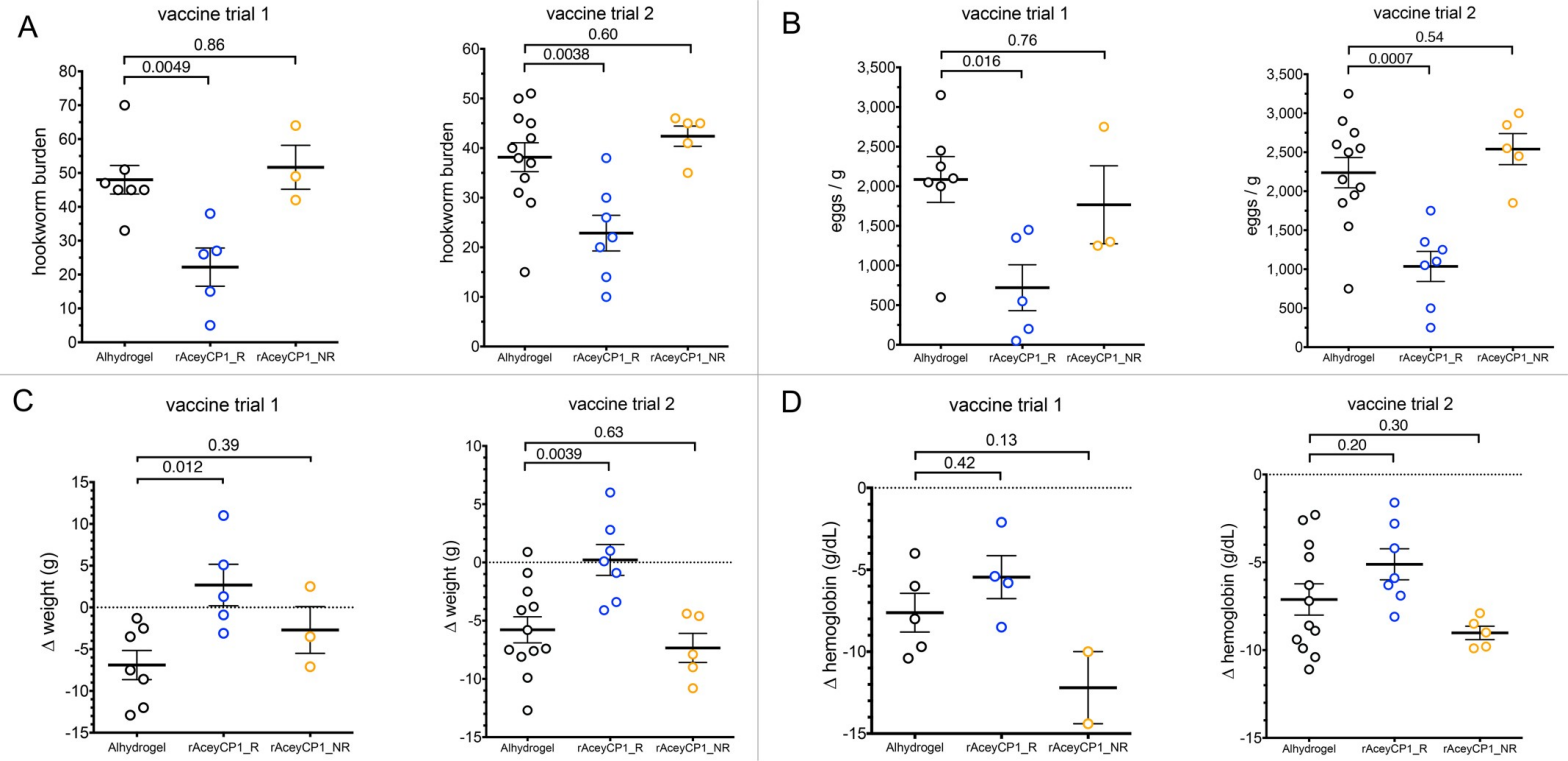
Efficacy results for rAceyCP1 serum IgG responders and nonresponders analyzed as separate groups. Comparisons between rAceyCP1/Alhydrogel serum IgG responders (blue dots) and serum IgG nonresponders (orange dots) with Alhydrogel controls (black dots) of (A) mean hookworm burdens, (B) mean FEC, (C) mean change in weight after hookworm infection and (D) mean change in hemoglobin concentration after hookworm infection R, responders; NR, nonresponders.

In both vaccine trials, we noticed that rAceyCP1 serum IgG responders were qualitatively more protected than nonresponders (Fig 6; compare blue and orange data points). We hypothesized that the responders were protected from infection and sequelae whereas the nonresponders were not. Thus, we reanalyzed them as separate groups, and compared them to Alhydrogel controls. Strikingly, in trial 1, hookworm burden was dramatically and significantly decreased in responders by 54% compared to Alhydrogel controls (Fig 7A), whereas nonresponders showed no protection compared to Alhydrogel controls (Fig 7A). In trial 2, hookworm burden was dramatically and significantly decreased in responders by 40% compared to Alhydrogel controls (Fig 7A), whereas nonresponders showed no protection compared to Alhydrogel controls (Fig 7A). Protection from infection based on hookworm burdens was mirrored in FECs. In trial 1, FECs were dramatically and significantly decreased in responders by 66% compared to Alhydrogel controls (Fig 7B), whereas nonresponders showed no protection compared to Alhydrogel controls (Fig 7B). Accordingly, in trial 2, FECs were dramatically and significantly decreased in responders by 54% compared to Alhydrogel controls (Fig 7B), whereas nonresponders showed no protection compared to Alhydrogel controls (Fig 7B). Sequelae based on Δweight were also improved. In trial 1, responders had a dramatically and significantly improved Δweight of 9.6 g compared to Alhydrogel controls (Fig 7C), whereas nonresponders showed no improved Δweight compared to Alhydrogel controls (Fig 7C). In trial 2, responders had a dramatically and significantly improved Δweight of 6.0 g compared Alhydrogel controls (Fig 7C), whereas nonresponders showed no improved Δweight compared to Alhydrogel controls (Fig 7C). In trials 1 and 2, responders had improved Δhemoglobin by 2.2 g/dL and 2.0 g/dL respectively, although neither was statistically significant (Fig 7D).

**Fig 8:**
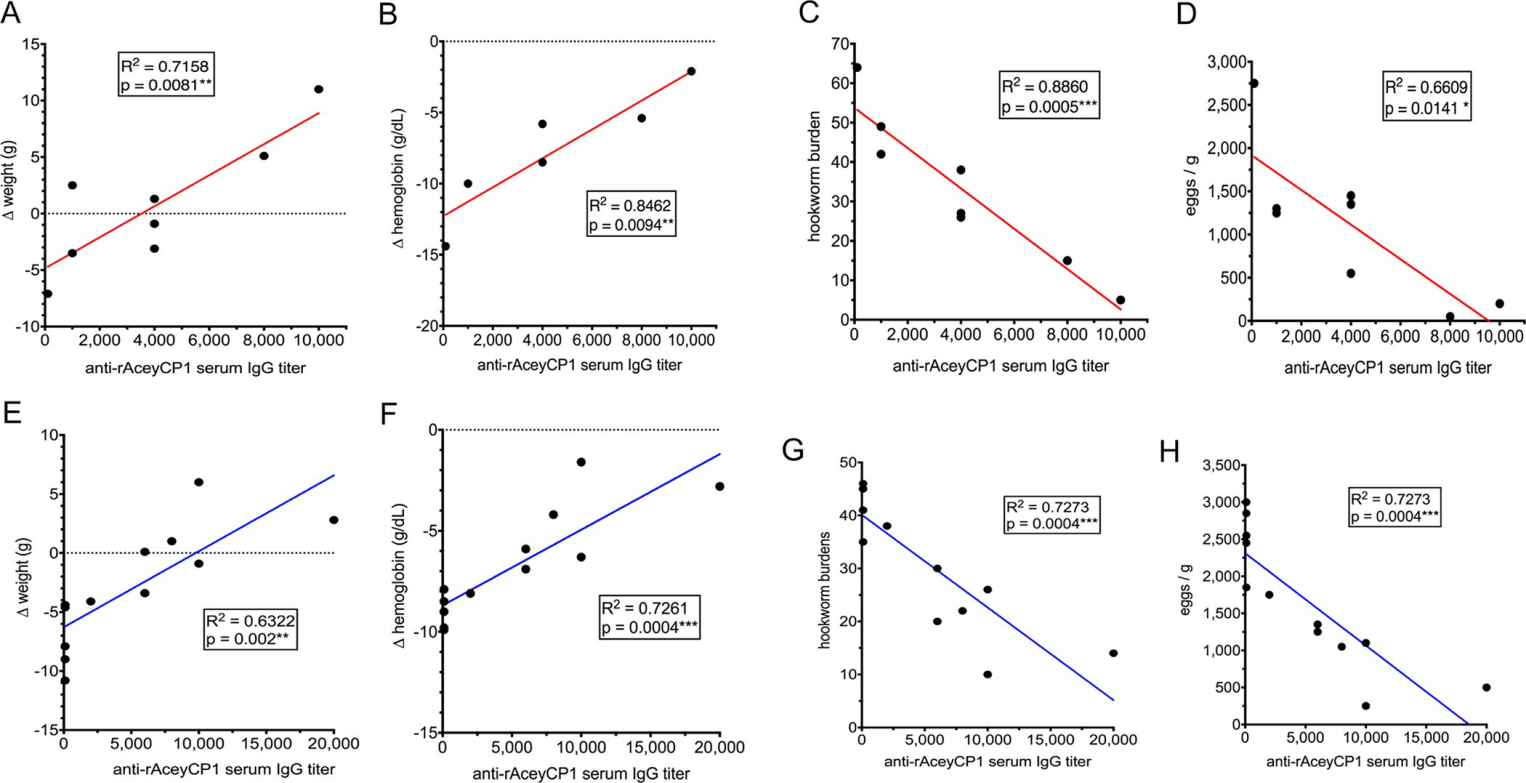
Linear regression of two measures of sequelae and two measures of infection status by rAceyCP1 serum IgG titer. (A-D) Vaccine trial 1. (E-H) Vaccine trial 2.

A complete summary of the efficacy results from rAceyCP1/Alhydrogel vaccine trials 1 and 2 is given in Table 1, with the two immunogens currently being tested in phase 1 clinical trials included for comparisons. Linear regression analysis was performed on the data to investigate the correlation between rAceyCP1 serum IgG titer and changes in sequelae and parameters of infection. These analyses determined that, in both vaccine trials, rAceyCP1 serum IgG titer highly significantly correlated with all four measures of protection, including Δhemoglobin (Fig 8). Moreover, serum IgG titers of 10,000–20,000 in the two trials (n = 4) gave a mean of 64.6% decreased hookworm burden and 76.9% decreased FECs compared to Alhydrogel controls. These findings indicate that when rAceyCP1 serum IgG titer is sufficiently induced, rAceyCP1/Alhydrogel is a highly protective hookworm vaccine.

**Table 1:** Summary of the mean efficacy results from rAceyCP1/Alhydrogel vaccine trials and comparison to clinical hookworm antigen candidates.

vaccine group	compared to adjuvant	Change in burden	Change in FEC	Change in weight (g)	Change in Hg (g/dL)
rAceyCP1_1—all	Alhydrogel	-30.7%*	-46.7%*	+7.6**	-0.1
rAceyCP1_1—R	Alhydrogel	-53.8%**	-65.5%*	+9.6*	+2.2
rAceyCP1_2—all	Alhydrogel	-18.8%	-25.7%*	+2.8	+0.4
rAceyCP1_2—R	Alhydrogel	-40.1%**	-53.7%***	+6.0**	+2.0
rAcanAPR1—R^a^	ASO3	-33.0%	-70.0%*	n/a	+3.0*
rAcanGST1—R^b^	ASO3	-39.4%	-32.3%	n/a	n/a

* P < 0.05.

** P < 0.01.

*** P < 0.001; No asterisk indicated not significant at P<0.05.

^a^Loukas et al. [27] vaccine trial in beagles; only published trial that thoroughly reported efficacy (hookworm burden, fecal egg count [FEC] and a measure of clinical pathology), and immunogenicity for APR1. rAcanAPR1 gave 100% responder (R) rate with serum IgG2 titer of ~121,500 and IgG1 titer of ~13,500.

^b^Zhan et al. [26] vaccine trial in beagles; only published trial that reported multiple measures of protection (hookworm burden and FEC), and immunogenicity for GST1. rAcanGST1 gave 100% responder rate with serum IgG2 titer of ~40,500 and IgG1 titer of ~13,500. For comparisons, rAceyCP1 responders gave serum IgG titers of only 4,000–10,000 in trial 1, and only 2,000–20,000 in trial 2. Additional abbreviations: Hg: Hemoglobin; R, responders; NR, nonresponders; Acan, *A*. *caninum*.

### Antisera from rAceyCP1/Alhydrogel responders significantly reduces adult hookworm motility *in vitro*

**Fig 9:**
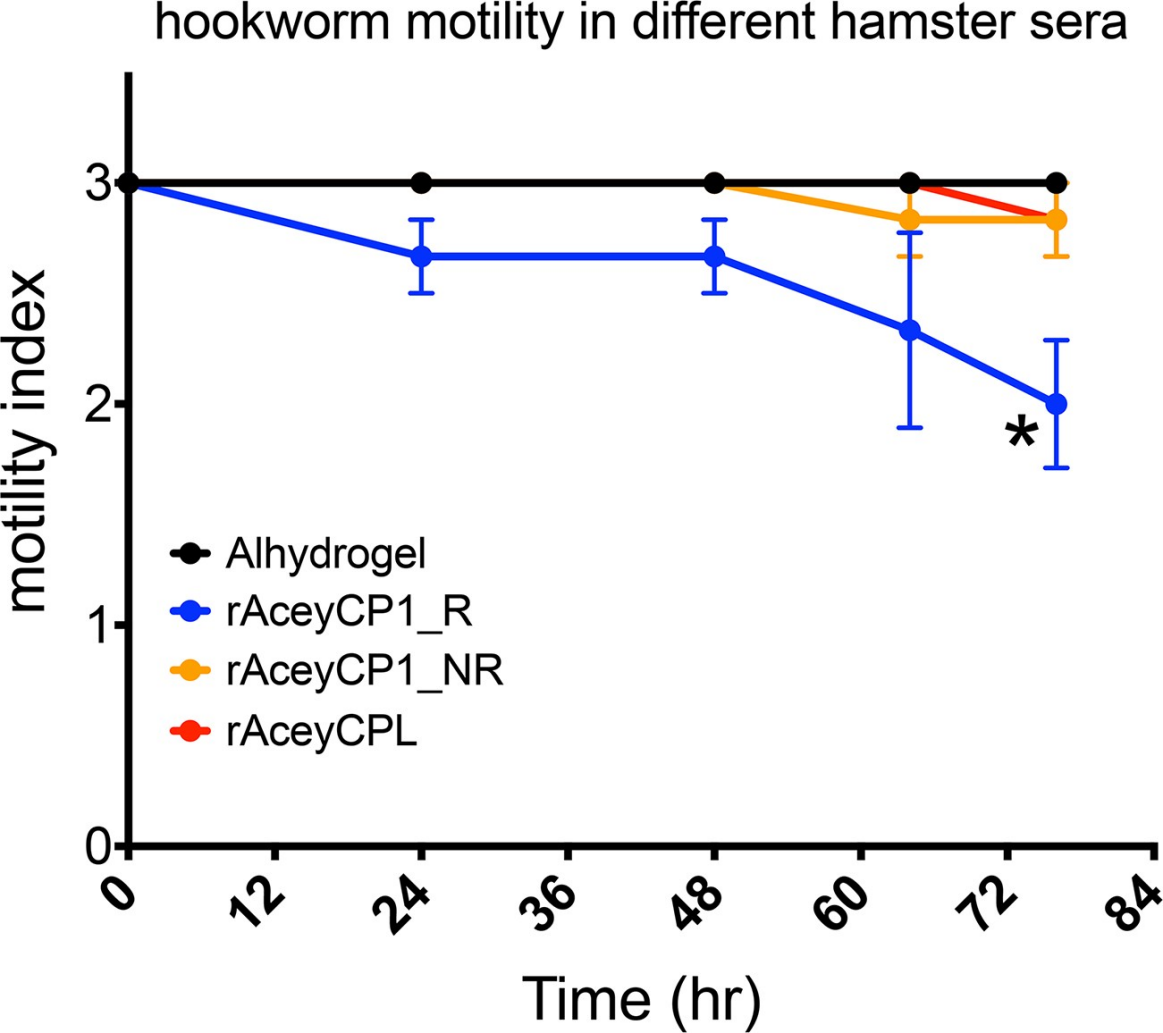
Antisera specifically from rAceyCP1/Alhydrogel IgG responders reduce adult hookworm motility *in vitro*. Motility of hookworms in different hamster sera over time. Adult *A*. *ceylanicum* hookworms were collected from naïve (unvaccinated) hamsters and incubated for 76 h in different hamster sera. Motility was scored using a motility index (see Materials and Methods). Shown is the mean motility and standard error. * P = 0.013.

We hypothesized that serum IgG might be at least partly responsible for vaccine-induced protections in rAceyCP1/Alhydrogel serum IgG responders. As a first test for this hypothesis, we incubated adult *A*. *ceylanicum* hookworms obtained from naïve hamsters in a modified hookworm culture medium (mHCM) containing 50% antisera from rAceyCP1/Alhydrogel serum IgG responders and nonresponders, as well as rAceyCPL/Alhydrogel vaccinates and Alhydrogel controls. We scored motility over a period of 76 hr; scoring was performed blind relative to treatment condition to prevent bias. Hookworm motility was significantly reduced by 76 hr in rAceyCP1/Alhydrogel serum IgG responder antisera by a mean of 33.3% compared to in Alhydrogel control sera (Fig 9). Conversely, hookworm motilities in serum IgG non-responder antisera and rAceyCPL/Alhydrogel antisera were reduced by just 5.6% and were statistically similar to Alhydrogel control sera (Fig 9), demonstrating specificity of the activity seen above. Thus, the unique toxicity of antisera alone from rAceyCP1/Alhydrogel serum IgG responders is consistent with the observed protections.

### rAceyCP1/Alhydrogel induces antigen-specific Th2 cytokines in serum IgG responders

Vaccination with rAceyCP1/Alhydrogel induced protection that was highly correlated with serum IgG titer, and found to intoxicate/reduce adult hookworm motility *in vitro*. In order to gain an understanding of the adaptive, cellular immune responses induced in rAceyCP1/Alhydrogel vaccinates, eight hamsters were vaccinated with rAceyCP1/Alhydrogel exactly as in trials 1 and 2. Peripheral blood was collected one week after the final immunization to measure serum IgG responses. Necropsy was performed exactly two weeks after the final immunization (*i*.*e*., at the exact time vaccinates were infected with L3i in trials 1 and 2), and splenocyte suspensions were prepared. rAceyCP1/Alhydrogel and Alhydrogel control splenocytes were stimulated *ex vivo* with rAceyCP1 or PBS. Total RNA was then isolated and used directly as template for quantitative real-time reverse transcription (qRT)-PCR using pre-validated primer sets [48] for the following cytokines: IFN-γ (Th1); IL4, IL5, IL13 (Th2); IL17A (Th17); IL21 (Th17/Tfh); IL10, TGF-β (Treg) (S1 Fig).

**Fig 10:**
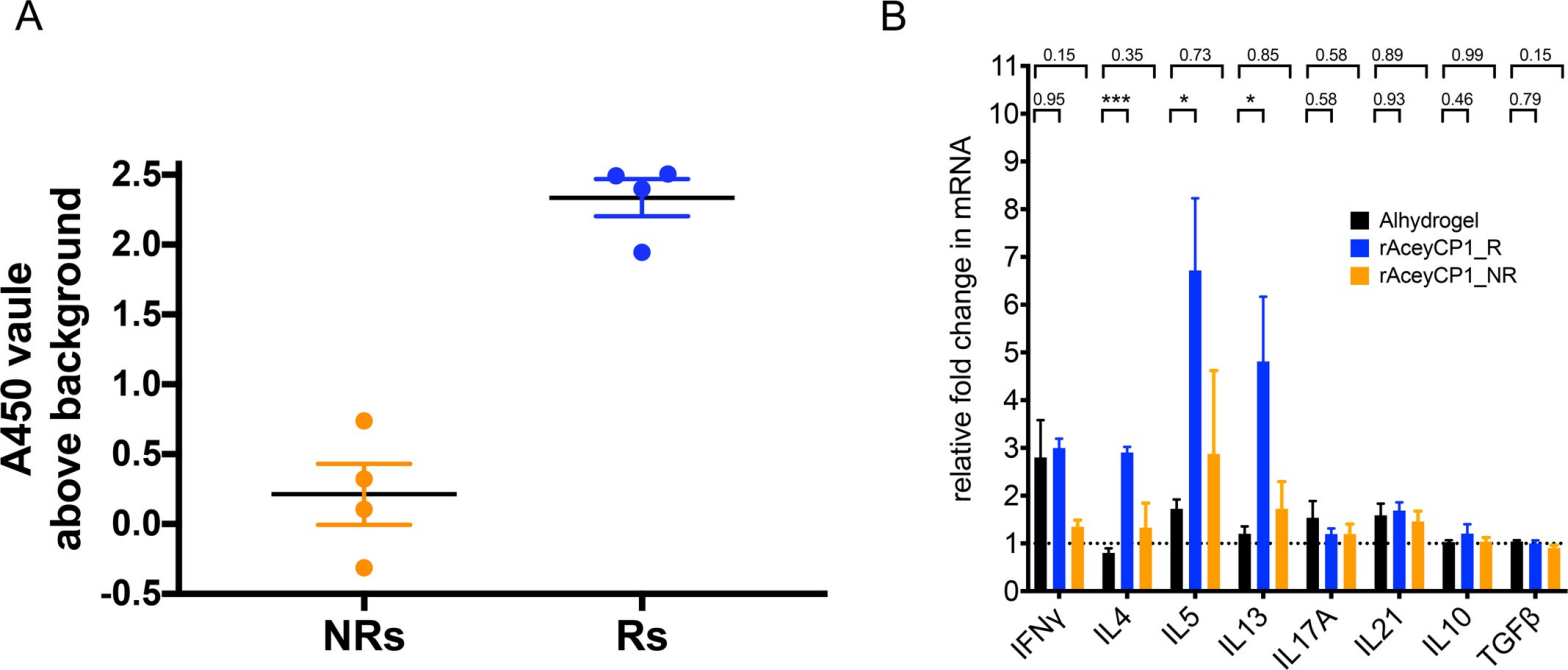
Assessment of rAceyCP1 splenic cytokine recall responses. (A) Serum IgG ELISA for rAceyCP1/Alhydrogel vaccinates. Reported are the raw absorbances at 450 nm (A450) using 1:100 serum dilutions with background subtracted (average absorbance of Alhydrogel alone). Orange dots indicate rAceyCP1 serum IgG nonresponders (NRs). Blue dots indicate rAceyCP1 serum IgG responders (Rs). (B) qRT-PCR of cytokine mRNA transcripts in total RNA isolated from splenocytes collected from Alhydrogel controls (black bars; n = 5), rAceyCP1/Alhydrogel serum IgG responders (R) (blue bars; n = 4 see (A)), and rAceyCP1/Alhydrogel serum IgG nonresponders (NR) (orange bars; n = 4 see (A)). Splenocytes were stimulated with rAceyCP1 or PBS. Reported are the mRNA fold changes in rAceyCP1 relative to PBS stimulated. Data were normalized to actin. *** P = 0.0007; * P = 0.028 for IL5 Alhydrogel vs rAceyCP1_R, P = 0.015 for IL13 Alhydrogel vs rAceyCP1_R.

rAceyCP1/Alhydrogel vaccinates (4/8) gave rAceyCP1 serum IgG responses (raw A450 readings) above background in Alhydrogel controls in serum diluted 1:100 (Fig 10A). rAceyCP1-stimulated splenocytes from rAceyCP1/Alhydrogel IgG responders (n = 4) resulted in elevated levels of all three Th2 cytokine mRNAs (IL4, IL5 and IL13) that were significantly greater than in stimulated splenocytes from Alhydrogel controls (n = 5; Fig 10B). No other cytokines were elevated in splenocytes from rAceyCP1/Alhydrogel serum IgG responders at levels that were significantly greater than in Alhydrogel control, indicating a highly specific, canonical Th2 cytokine recall response. Also, all cytokines in stimulated splenocytes from rAceyCP1/Alhydrogel IgG nonresponders were statistically similar to Alhydrogel control (Fig 10B).

## Discussion

We demonstrate here that the highly expressed, substantially intestine-enriched cathepsin B cysteine protease, AceyCP1, is a promising protective antigen candidate for vaccination against *A*. *ceylanicum* hookworm infection in Syrian hamsters. Two antigens (AceyCPL, AceySKPI3) with much lower transcript levels in the adult *A*. *ceylanicum* intestine compared to AceyCP1 were not protective (Figs 3 and 4). These data are supportive of the value of using intestinal expression to prioritize antigen candidates.

Other intestinal CPs were shown to be protective against other blood-feeding gastrointestinal nematodes (canine hookworm *A*. *caninum*, small ruminant parasite *H*. *contortus*, *N*. *americanus* hookworms) in animal hosts (dogs, sheep, hamsters respectively) [29,55–58], albeit not to the extent that we report here for AceyCP1 (*e*.*g*., vaccination with *N*. *americanus* CP2 gave 29% reduction in worm burdens in hamsters). Interestingly, intestinal CPs were shown not to be protective against an STN parasite of cattle that does not ingest blood, *Ostertagia ostertagi* [59,60], whereas CPs in *O*. *ostertagi* ES products were shown to be protective [59,61]. Moreover, recently, IgG induced by vaccination with whole worm extracts of *Ascaris suum* (another non-blood-feeding STN) was determined (in IgG transfer experiments) to be protective against infection [62]. Furthermore, *O*. *ostertagi* intestinal CP components in a larger native protein complex did cross-protect against *H*. *contortus* in vaccinated sheep [60]. These important CP enzymes are therefore vulnerable antigens that can be accessed in the gut only by immune factors that are ingested in the blood. Previous studies of intestinal CPs of blood-feeding STNs have localized the CPs within the worm intestinal lumen with serum IgG from vaccinated hosts, and serum IgG has been implicated as the effector component that neutralizes CP digestion of the blood meal [55,56,58,63,64]. However, our finding that AceyCPL vaccination gave a strong immune response but no protection confirms that not all cathepsin B cysteine protease antigens are useful for vaccination.

rAceyCP1/Alhydrogel serum IgG responders had dramatically decreased hookworm burdens, FECs, and weight losses in two different vaccine trials compared to Alhydrogel controls, whereas nonresponders did not (Fig 7, Table 1). rAceyCP1/Alhydrogel is a highly protective vaccine, reducing hookworm burdens and FECs by ~50% and ~60%, respectively, in responders (Fig 7, Table 1), and ~65% and ~77%, respectively, when serum IgG titers were ≥10,000. Although there were moderately decreased blood losses in responders compared to Alhydrogel controls (Fig 7, Table 1), these results were not significant. On the other hand, blood loss was highly negatively correlated with rAceyCP1 serum IgG titer (Fig 8).

rAceyCP1-induced protection is among the highest seen to date of current hookworm antigens (Table 1). Consistently, *AceyCP1* is expressed 220 and 6,800 times more strongly in the *A*. *ceylanicum* intestine compared to *AceyAPR1* and *AceyGST1* (11,600 TPM versus 52 and 1.7 TPM, respectively) (Table S2). Furthermore, *AceyCP1* is almost completely unexpressed in L3i (0.15 TPM), while *AceyAPR1* and *AceyGST1* have significant expression levels in L3i (744 and 44 TPM, respectively). Thus, AceyCP1 is unlikely to be recognized by IgE and to induce urticarial reactions in previously exposed people from hookworm endemic regions [24], since L3i is the predominant IgE-reactive stage [65]. Although we cannot rule out effects from different adjuvants, hosts, and *Ancylostoma* species of vaccine studies carried out to date, rAceyCP1 is clearly a positive addition to the hookworm vaccine antigen arsenal.

Antisera from rAceyCP1/Alhydrogel responders, but not from nonresponders or rAceyCPL/Alhydrogel vaccinates, reduced adult *A*. *ceylanicum* motility *in vitro* as early as 24 hr, and was significant by 76 hr (Fig 9). These results are consistent with a model whereby neutralizing serum IgG inhibit AceyCP1 blood digestion within the hookworm gut, thus leading to starvation—comparable to models for other gut protease antigens of blood-feeding STNs [25,27,56,58,63].

We observed Th2-specific cytokine recall responses in stimulated splenocytes from rAceyCP1 responders *ex vivo* that highly correlated with serum IgG titer (Fig 10). Our findings reinforce the notion that neutralizing IgG in serum (likely assisted by Th2 cytokines) is the protective component to gut antigens in blood-feeding STNs.

Vaccinated hamsters in trials 1 and 2 gave ~60% responder rates (Fig 5). Clearly, a major focus in the future will be to improve responder rates, and rAceyCP1 has the potential to teach us more about how to make a better hookworm vaccine. The ~40% nonresponder rate could be due to a number of factors. First, rAceyCP1 was expressed as a secreted, partially glycosylated protein in *P*. *pastoris* yeast consisting of unglycosylated, monoglycosylated and biglycosylated forms with relative abundances as follows: biglycosylated > unglycosylated > monoglycosylated (Fig 1A–1C). Glycosylation of rAceyCP1 may play an important role in the IgG nonresponder rate and/or intermediate titers in responders. It has been hypothesized that proper antigen glycosylation plays a critical role in vaccine efficacy of antigens against other gastrointestinal nematode parasites (reviewed in [66]). Expressing rAceyCP1 in other systems with other glycosylation patterns is therefore an important next step. Another potential contributing factor to the ~40% nonresponder rate and intermediate rAceyCP1 serum IgG titers in responders is major histocompatibility complex (MHC) class II (MHC-II) allelic variation in the outbred hamster colony, which would give variable Th cell responses [67]. Envigo maintains their hamster colony as outbred by non-sib matings and claims that due to high litter average and reproductive vigor, there should be a diversity of MHC alleles segregating in the colony.

In conclusion, rAceyCP1/Alhydrogel is a highly protective vaccine against *A*. *ceylanicum* hookworm infection in Syrian hamsters when serum IgG is sufficiently induced, which likely requires help from Th2 cytokines. Serum IgG may target AceyCP1 within the gut, thereby neutralizing hookworm digestion of the blood meal. Efforts are underway to improve rAceyCP1 neutralizing IgG responder rate and titers, efforts that will ultimately improve translation to humans. Hookworm is among the most disabling parasitic diseases of the developing world, and our findings provide important information for advancing hookworm vaccine development.

## Supporting information

S1 Table Primers. [*]Published by Espitia et al. [48]; primers sets for the other cytokines had to be redesigned and validated by us (S1 Fig), because Espitia et al. [48] primer sets did not work for our sample type.
(DOCX)

S2 Table Protein-coding gene predictions, annotations, and RNA-seq values for *A*. *ceylanicum* genes. Included as Separate Excel Table. Data columns are as follows. **Gene:** a given predicted protein-coding gene in the *A*. *ceylanicum* genome assembly [35]. All further data columns are pertinent to that particular gene. **Vaccine candidate:** specific abbreviations used in this text for genes encoding possible subunits of vaccines (e.g., AceyCP1 for the gene Acey_s0154.g3007). **Annotations.** Most of these annotations are from our previous analyses [35]. However, two sets of annotations have been updated and recomputed for this paper: Pfam and InterPro. **Coordinates:** genomic coordinates in the *A*. *ceylanicum* genome assembly. The strand orientation of the gene, with respect to the assembly sequence's orientation, is given as '[+]' (sense strand) or '[–]' (antisense strand). **Max_prot_size:** the size of the largest predicted protein product. **Start/stop codons:** whether a predicted gene has both start and stop codons ('Complete'), a start or stop codon only ('Start_codon' or 'Stop_codon'), or is a completely fragmentary prediction (blank in this field; there are 9 such genes, all of which come from smaller genomic scaffolds). **Prot_size:** this shows the full range of sizes for all protein products from a gene's predicted isoforms. **Phobius:** this denotes predictions of signal and transmembrane sequences made with Phobius [68]. 'SigP' indicates a predicted signal sequence, and 'TM' indicates one or more transmembrane-spanning helices, with N helices indicated with '(Nx)'. Varying predictions from different isoforms are listed. **Psegs:** this shows what fraction of a protein is low-complexity sequence, as detected by pseg [69]. Both the proportion of such sequence (ranging from 0.01 to 1.00) and the exact ratio of low-complexity residues to total residues are given. Proteins with no predicted low-complexity residues are blank. **NCoils:** this shows coiled-coil domains, predicted by ncoils [70]. As with Psegs, the relative and absolute fractions of each protein's coiled-coil residues are shown. **Pfam:** newly predicted domains (in this study) from Pfam 31.0 [71], using *hmmscan* with reliably curated domain-specific thresholds. **InterPro:** newly predicted domains (in this study) from InterPro [72], using InterProScan 5.18–57.0 with default settings. **GO_terms:** Gene Ontology terms, generated with Blast2GO using default settings. **Housekeeping:** a set of 406 genes (with strict, 1-to-1 orthologies between the two species being compared) that we observed to be consistently active both in *A*. *ceylanicum* and *C*. *elegans* under all RNA-seq conditions tested. These conditions included a complete passage through infection for *A*. *ceylanicum*, and a complete life cycle determined by modENCODE for *C*. *elegans*. **ASP:** 432 genes that we designated as being classic ASP genes, as defined by their having at least one copy of a PFAM CAP domain (PF00188.21) at E ≤ 10^−2^, and by their not having already been defined as ASPR genes (this second condition excluded only one gene). This definition successfully detected all previously known ASPs in *Ancylostoma*, including the highly divergent ASP-7. **ASPR:** 92 genes that we designated as a cryptic ASP-Related gene family, on the basis of strong similarities to one another (as defined by a convergent psi-BLAST search with an E-value threshold of 10^−9^) and by more distant similarities to ASP genes (detectable either with psi-BLAST or with interative HMMER/jackhmmer searches). **SL4P:** 24 genes that we designated as a family of Strongylid L4 Proteins, on the basis of strong similarities to one another (as defined by a convergent psi-BLAST search with an E-value threshold of 10^−6^). **SCVP:** 53 genes that we designated as a family of Secreted Clade V Proteins, on the basis of strong similarities to one another (as defined by a convergent psi-BLAST search with an E‑value threshold of 10^−6^). **Other_secreted_groups:** the purpose of this category was to detect small, secreted, and conserved but unfamiliar proteins that might be up- or down-regulated during infection. This general category includes subcategories whose statistical overrepresentations during changes of gene activity (between developmental stages or drug treatments) are listed in Supplementary Table 10. The subcategories are: Secreted_100, Secreted_150, Secreted_200, Secreted_Any, Cons_Secreted_100, Cons_Secreted_150, Cons_Secreted_200, Cons_Secreted_Any, Hco_Cons_Secreted_100, Hco_Cons_Secreted_150, Hco_Cons_Secreted_200, Hco_Cons_Secreted_Any, Hco_Cons_Only_Secreted_100, Hco_Cons_Only_Secreted_150, Hco_Cons_Only_Secreted_200, and Hco_Cons_Only_Secreted_Any. To be in any of these subcategories, a gene had to have a full-length gene prediction (i.e., not be fragmentary). To be 'Secreted', a gene had to have at least one product predicted to be classically secreted by Phobius; to be 'Cons_Secreted', it also had to belong to an OrthoMCL group from the 14-species OrthoMCL run; to be 'Hco_Cons_Secreted', the OrthoMCL group had to include *H*. *contortus*; to be 'Hco_Cons_Only_Secreted', the OrthoMCL group had to include only *A*. *ceylanicum* and *H*. *contortus*. For products with maximum sizes of ≤100, ≤150, or ≤200 residues, these sizes are noted as suffixes. **Acey-specific_protease:** a set of protease genes that we identified as being of particular interest as vaccine candidates due to their being upregulated during early infection, not being significantly downregulated, not having mammalian orthologs, and having one or more *H*. *contortus* homologs that were also upregulated during infection. **Acey-specific_protease_inhibitor:** a single protease inhibitor gene selected as a possible vaccine candidate, by identical criteria to those used for proteases. **Drug_target:** a set of 80 genes whose products we identified as being of particular interest as drug targets, based upon a visible phenotype in *C*. *elegans*, a known homologous protein structure, presence in one or more other parasites, absence from mammals, and a known small-molecular ligand (ideally, a drug). On manual examination, we rejected 8 of these genes as being likely to be mispredictions (because BlastP or psi-BLAST against NCBI-nr revealed them to have mammalian homologs) but accepted 72 others as being plausible drug targets. All 80 genes are annotated with their protein class and any information about their feasibility as drug targets. RNA-seq expression values. All of these have been computed for this paper as described in Methods. The data for adult male intestine are from Mitreva and coworkers [38]; all other RNA-seq data are from our previous work [35]. By recomputing all of these data in a single pipeline, we generated expression values that could be reasonably compared between the two publications. **Acey_[stage].TPM, for the following stages—male_intestine, L3i, 24.HCM, 24.PI, 5.D, 12.D, 17.D, 19.D, Alb.4hr.D18, noAlb.cont.D18, 18D.Cry5B.4hr, 18D.HEPES.4hr, 18D.Cry5B.24hr, 18D.HEPES.24hr, 18D.SB.plusCry5B, 18D.SB.plusHEPES:** the gene expression levels detected in each of these stages, in units of Transcripts Per Million (TPM), as computed by Salmon [40]. **Acey_[stage].reads, for the following stages—male_intestine, L3i, 24.HCM, 24.PI, 5.D, 12.D, 17.D, 19.D, Alb.4hr.D18, noAlb.cont.D18, 18D.Cry5B.4hr, 18D.HEPES.4hr, 18D.Cry5B.24hr, 18D.HEPES.24hr, 18D.SB.plusCry5B, 18D.SB.plusHEPES:** the estimated number of reads (rounded to the nearest integer) mapped to the genome for a given gene, as computed by Salmon [40]. For both TPM and reads, the [stage] abbreviations signify the following stages. male_intestine: dissected adult male intestines [38]. L3i: Infectious L3 larvae. 24.HCM: L3i incubated for 24 hr in hookworm culture medium simulating infection. 24.PI: Larvae 24 hours after infection (as L3i), in early stages of infection; majority of worms in stomach. 5.D: Larvae 5 days post infection (d.p.i.); sexual differentiation distinct, and majority of larvae in final L4; worms in small intestine until adulthood. 12.D: Worms 12 d.p.i.; early adult stage, with few gravid females and mature males. 17.D: Worms 17 d.p.i.; start of mature adult stage with onset of egg-laying. 19.D: Worms 19 d.p.i.; mature adult stage. Alb.4hr.D18: Worms 18 d.p.i. treated with albendazole for 4 hr. noAlb.cont.D18: Worms 18 d.p.i. treated with 0.2% DMSO buffer rather than albendazole for 4 hr. 18D.Cry5B.4hr: Worms 18 d.p.i., then exposure to 100ug/mL Cry5B for 4 hr. 18D.HEPES.4hr: Worms 18 d.p.i., then HEPES buffer control for 4hr. 18D.Cry5B.24hr: Worms 18 d.p.i., then worms incubated in hookworm culture media for 24 hrs, then exposed to 100ug/mL Cry5B for 4 hr. 18D.HEPES.24hr: Worms 18 d.p.i., then worms incubated in hookworm culture media for 24 hrs, then exposed to HEPES buffer control for 4 hr. 18D.SB.plusCry5B: Worms 18 d.p.i., then worms incubated in hookworm culture media with 50uM SB203580 (p38 MAPK inhibitor) for 24 hrs, then exposed to 100 ug/mL Cry5B for 4 hr. 18D.SB.plusHEPES: Worms 18 d.p.i., then worms incubated in hookworm culture media with 50uM SB203580 for 24 hr, then exposed to buffer control for 4 hr.
(XLSX)

S1 Fig qRT-PCR primer efficiency tests for Syrian hamster cytokine primer sets on splenocyte total RNA. Shown are threshold cycle (Ct) values regressed by Log10 serial dilutions. Amplification factors and efficiencies were calculated using qPCR Efficiency Calculator (Thermo Fisher).
(TIF)
