# Supplementary material for: A highly expressed intestinal cysteine protease of Ancylostoma ceylanicum protects vaccinated hamsters from hookworm infection
Source: PLoS Negl Trop Dis. 2019 Apr 22;13(4):e0007345. doi: 10.1371/journal.pntd.0007345 (PMC6497320; doi:10.1371/journal.pntd.0007345)
Supplement: S1 Table — [*]Published by Espitia et al. [48]; primers sets for the other cytokines had to be redesigned and validated by us (S1 Fig), because Espitia et al. [48] primer sets did not work for our sample type. (DOCX) [file pntd.0007345.s001.docx]

**Table S1. Primers.**

| **Primer ID** | **Sequence (5’-3’)** |
| --- | --- |
| Aceys0154g3007cdsF | ATGTGGATCCTCGCAGCGTTATTG |
| Aceys0154g3007cdsR | TCACACTCTCATGATTCCACCAACCAT |
| Aceys0532g3038t1cdsF | ATGATCGTCATCCTAACACTTCTTACGG |
| Aceys0532g3038t1cdsR | CTAGACCTTCATCATGCCAGCG |
| Aceys0034g2829t1cdsF | ATGAGCCTCGCCTTCTACC |
| Aceys0034g2829t1cdsR | CTACTTTTCACAAGCTTTTTGACACTCT |
| Ma_G-ACTIN_F* | ACAGAGAGAAGATGACGCAGATAATG |
| Ma_G-ACTIN_R* | GCCTGAATGGCCACGTACA |
| Ma_IFN-γ_F* | TGTTGCTCTGCCTCACTCAGG |
| Ma_IFN-γ_R* | AAGACGAGGTCCCCTCCATTC |
| Ma_IL-4_F2 | CTTCTAGCATGTACCGGGAACTGGA |
| Ma_IL-4_R2 | GAAAGGGCGTCTGGTACGACC |
| Ma_IL-5_F1 | GGCTTCCTGTTCCCACTCATAA |
| Ma_IL-5_R1 | GTTTTGGAATAGCGTCTCCACG |
| Ma_IL-13_F* | AAATGGCGGGTTCTGTGC |
| Ma_IL-13_R* | AATATCCTCTGGGTCTTGTAGATGG |
| Ma_IL-17A_F3 | GTTCTCATCCAGCAAGAGATCCTGGT |
| Ma_IL-17A_R3 | AATGGAGGAAACGCAGGTGCAG |
| Ma_IL-21_F* | GGACAGTGGCCCATAAAACAAG |
| Ma_IL-21_R* | TTCAACACTGTCTATAAGATGACGAAGTC |
| Ma_IL-10_F* | GGTTGCCAAACCTTATCAGAAATG |
| Ma_IL-10_R* | TTCACCTGTTCCACAGCCTTG |
| Ma_TGF-ß1_F* | GGCTACCACGCCAACTTCTG |
| Ma_TGF-ß1_R* | GAGGGCAAGGACCTTACTGTACTG |

[*]Published by Espitia et al. [[48]](https://paperpile.com/c/yk9gDc/ytaim); primers sets for the other cytokines had to be redesigned and validated by us (Fig S1), because Espitia et al. [[48]](https://paperpile.com/c/yk9gDc/ytaim) primer sets did not work for our sample type.
