# Supplementary figures and images for: A highly expressed intestinal cysteine protease of Ancylostoma ceylanicum protects vaccinated hamsters from hookworm infection
Source: PLoS Negl Trop Dis. 2019 Apr 22;13(4):e0007345. doi: 10.1371/journal.pntd.0007345 (PMC6497320; doi:10.1371/journal.pntd.0007345)

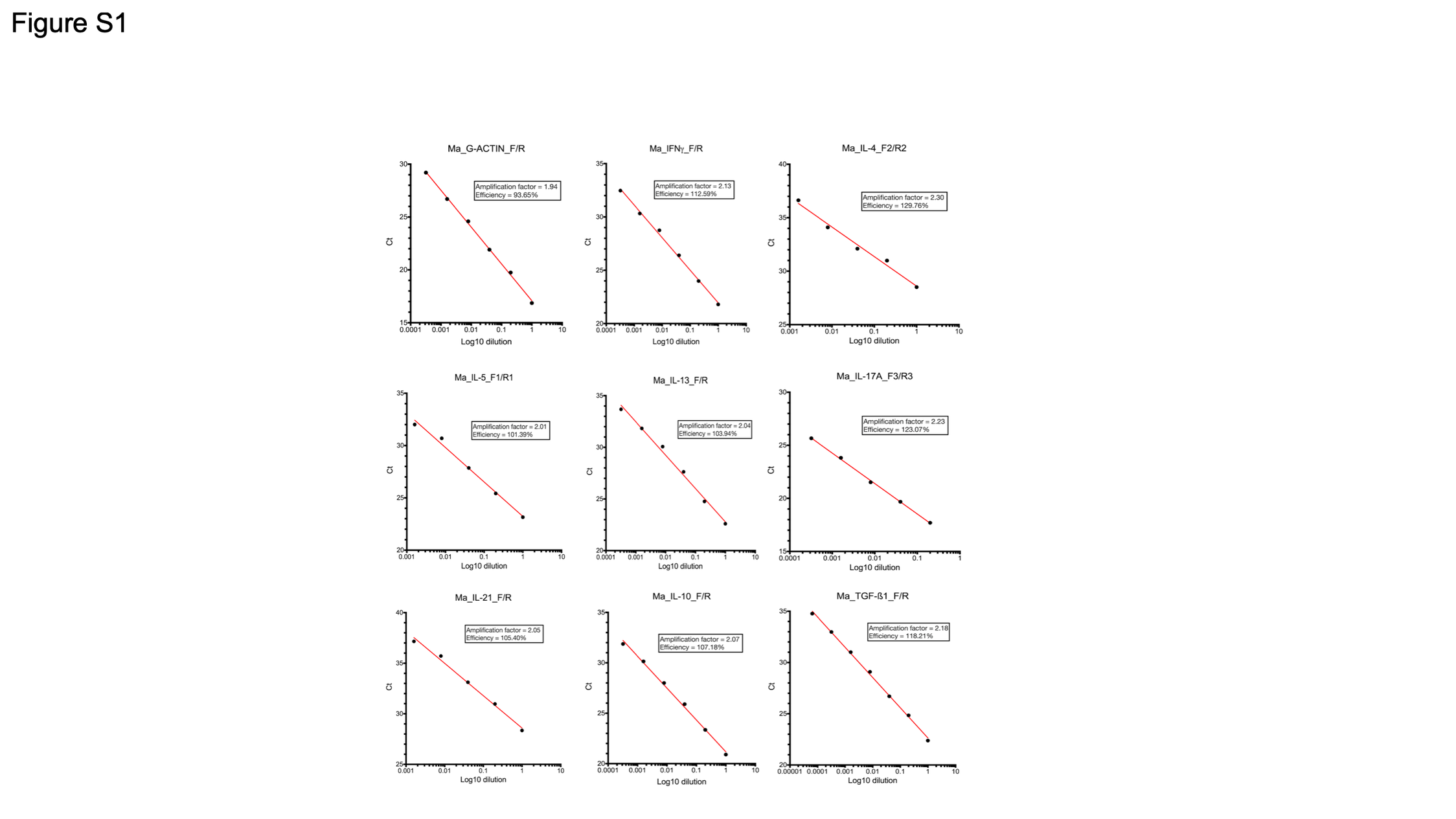

Supplement: S1 Fig — Shown are threshold cycle (Ct) values regressed by Log10 serial dilutions. Amplification factors and efficiencies were calculated using qPCR Efficiency Calculator (Thermo Fisher). (TIF) [file pntd.0007345.s003.tif]
